# Searching basic units in memory traces: associative memory cells

**DOI:** 10.12688/f1000research.18771.1

**Published:** 2019-04-12

**Authors:** Jin-Hui Wang

**Affiliations:** 1 College of Life Sciences, Chinese Academy of Sciences, Beijing, 100049, China

**Keywords:** Associative memory cell, synapse, neuron, learning, memory trace, cognition, brain

## Abstract

The acquisition of associated signals is commonly seen in life. The integrative storage of these exogenous and endogenous signals is essential for cognition, emotion and behaviors. In terms of basic units of memory traces or engrams, associative memory cells are recruited in the brain during learning, cognition and emotional reactions. The recruitment and refinement of associative memory cells facilitate the retrieval of memory-relevant events and the learning of reorganized unitary signals that have been acquired. The recruitment of associative memory cells is fulfilled by generating mutual synapse innervations among them in coactivated brain regions. Their axons innervate downstream neurons convergently and divergently to recruit secondary associative memory cells. Mutual synapse innervations among associative memory cells confer the integrative storage and reciprocal retrieval of associated signals. Their convergent synapse innervations to secondary associative memory cells endorse integrative cognition. Their divergent innervations to secondary associative memory cells grant multiple applications of associated signals. Associative memory cells in memory traces are defined to be nerve cells that are able to encode multiple learned signals and receive synapse innervations carrying these signals. An impairment in the recruitment and refinement of associative memory cells will lead to the memory deficit associated with neurological diseases and psychological disorders. This review presents a comprehensive diagram for the recruitment and refinement of associative memory cells for memory-relevant events in a lifetime.

## Introduction

Associative learning stands for a process, in which multiple exogenous signals, such as information, experiences and knowledge, are jointly acquired by sensory systems. Associative memory is termed as the integrative storage of these associated signals in the brain, which is indicated by memory retrievals (recall and representation) on the basis of speech, writing, gesture, countenance and emotion reactions. Associative learning and memory is a very common approach of signal storage for cognitions in life ^1– 6^ since the acquisition of new unitary signals or the reorganized learning of previously acquired unitary signals are fulfilled in the integrative manner ^7^. In learning processes, the associated exogenous signals come from sensory organs and reside into sensory cortices through cross-modal and intramodal manners ^7, 8^. The coactivation of sensory cortical neurons makes their axon projections and synapse innervations mutually, in which these neurons are recruited to be primary associative memory cells for the integrative storage and reciprocal retrieval of multiple exogenous signals ^9– 11^. In the meantime, these primary associative memory cells project their axons and make synapse innervations convergently onto their downstream neurons in certain brain areas for the integrative storage of endogenous associated signals that are essential to logical reasoning, associative thinking and other integrative cognitions. Their axons also divergently innervate the neurons in various brain areas that are relevant to cognition, emotion and behaviors for the storage of endogenous signals in many places and the participation in multiple memory-relevant events ^7, 12^. As cognitive processes and emotional reactions can be recalled, these neurons that memorize endogenous signals are named as secondary associative memory cells ^7, 8^. In other words, when the neurons encoding one of associated signals are activated, they can attract axon projection and synapse innervations from the coactivated neurons encoding another of associated signals. After associative memory cells are recruited from the coactivated neurons by receiving the innervation of multiple synapse inputs, their subsequent activities will change their excitability and synapse functions. The recruitment and refinement in the population of associative memory cells constitute a principle of activity together, connection together, strengthening together and coordination together ^7, 8^. Therefore, associative memory cells are nerve cells that are able to encode multiple signals which have been associatively acquired, as well as receive axon projection and synapse innervations that carry these signals. Currently, two kinds of associative memory cells have been detected experimentally, which carry out the activities of searching basic units of memory traces. Comprehensive cellular architectures underlying associative memory in the different periods of the lifespan are yet to be revealed to better understand memory-related physiology and psychology in the brain. This review focuses on current advances in associative memory cells as basic units of memory trace or engrams, an expansion on our previous review ^7^.

## Associative learning and memory

Learning is defined as the acquisition of new information, knowledge and experiences, which may be new unitary signals or the reorganized unitary signals that are learned previously. Signal acquisition can be classified into associative and non-associative styles ^1, 6^. Associative learning stands for the joint acquisition of multiple signals that can be sensory signals or sensory signals plus rewards after behavior operations are achieved. Their joint acquisitions are characterized as the association of new signals with an innate signal or a formerly learned signal, as well as the reorganized association of unitary signals that have been previously learned, such as the reorganization of letters into different words and phrases ^7^. Associative memory to these signals appears emerged if these associated signals can be retrieved reciprocally by cues or recalled by automatic conversion among sensory modalities. Two physiognomies of associative memory are the integrative storage and distinguishable retrieval of associated signals ^11^. On the other hand, non-associative learning is termed as the acquisition of a given sensory signal, in which repeated stimulations lead to habituation or sensitization to this sensory signal ^1^. It is thought that non-associative learning is not involved in acquiring new knowledge and experiences, except repetitive activations in a sensory system for its upregulation or downregulation by the sensitization or desensitization of sensory receptor and cortical neurons. In this regard, it may fall into the term “review” instead of learning. Therefore, the acquisition and storage of almost all of the new signals require them be associated with a signal that has been stored in the brain for the facilitation of their memory ^7^.

In associative learning, multiple featured signals in each object or an environment are detected by different modalities from sensory receptors to cerebral cortices. These cross-modal signals are integrated for their associative storage. For instance, a fruit is detected by the olfactory system for aromatic odor, the visual system for shape and color, the taste system for sweetness, the auditory system for name and the somatosensory system for its surface sign. After they are jointly memorized, a signal induces the recall of its associated signals, or the other way around. The recall can even be done by automatic signal conversion among different modalities, e.g., image signals are recalled by verbal signals with no additional cues. In addition, multiple signals with identical features can be associatively detected through a single modality, i.e., one type of sensory receptors and its projected cortex. These signals are associatively acquired in an intramodal manner and primarily stored in one of sensory cortices. Therefore, memory traces imprint the joint storage and distinguishable retrieval of multiple associated signals. Primary associative memory cells that encode multiple signals based on the synapses from innate inputs and new innervations from coactivated brain areas have been detected for the integrative storage of associated signals ^9– 11, 13– 17^.

In addition, logical reasoning, associative thinking, computation and integrative imagination based on those exogenous associated signals that have been stored in primary associative memory cells of sensory cortices may lead to the secondary integrations of those signals for their storage and representation, i.e., secondary associative memory that is essential for cognition, emotions and behaviors at the high orders under the consciousness condition ^7, 8^. Although the memory occurs presumably in the prefrontal cortex, hippocampus and amygdala ^18– 30^, these studies still do not reveal whether the memory in their data is secondary to the information storage in primary associative memory cells from sensory cortices. Currently, secondary associative memory cells are detected in the motor cortex, the prefrontal cortex and the hippocampus, which reside in the downstream of sensory cortices and receive synapse innervation from primary associative memory cells ^12, 31, 32^. 

Various memory patterns are classified in psychology, such as explicit versus implicit memory, declarative versus nondeclarative memory and episodic versus semantic memory in memory contents as well as sensory versus short-term or long-term memory in temporal feature ^6, 33^. Declarative memory, i.e., explicit memory, refers to stored information that can be stated consciously, including episodic memory (specific processes and their contexts) and semantic memory (generalized knowledge and concepts). Non-declarative memory, or implicit memory, denotes the operations of various skills and procedures without the need of consciousness. In fact, there is no clear border line between explicit and implicit memory. The procedures and skills operated in the implicit memory can be consciously stated. The specific processes and their contexts after repetitive practices can be executed effortlessly. In the field of neuroscience, the classifications of memory formation in the brain are based on the combination of the location of information storage with the memory of featured signals, for instance, spatial memory in the hippocampus, emotional memory in the amygdala, perceptual memory in sensory cortices and prospective, attentive and working memory in the frontal cortex ^6^. Moreover, it has been suggested that memory formation is classified based on cellular mechanisms, such as the different types of associative memory cells in the neural circuit for memory, i.e., memory trace or engram, and the sources of memorized signals from cross-modal versus intramodal sensory systems or exogenous versus endogenous resources ^7, 8^.

Associative memory to exogenous signals from external environments refers to the integration and storage of associated signals that are inputted from cross-modal or intra-modal sensory modalities. Associative memory to endogenous signals refers to the integration and the storage of associated signals that originate from sensory cortices and regenerate during logical reasoning and associative thinking in cognition- and emotion-relevant brain areas. Intramodal associative memory is related to the integration and storage of associated signals inputted from a single modality, such as a sensory modality or a brain area involved in cognition, emotion or behavior. Cross-modal associative memory is termed as the integration and storage of associated signals that come from different sensory modalities or brain regions related to cognitions, emotions or behaviors ^7, 8^.

To better understand these memory patterns and their correspondent mechanisms, we should figure out the basic units in memory traces or engrams that conduct the integration and storage of associated signals, constitute the foundation of cognitions (logical reasoning, associative thinking, computation, imagination and so on), achieve the integration and storage of endogenous signals generated from associative thinking and logical reasoning, and control the future presentation of stored associative signals. How the memory is formed in different modalities and encoded under the different states of consciousness, attention and psychological motion remains to be elucidated. Therefore, the comprehensive view of cellular mechanisms underlying associative memory should be established like an effort to see individual trees as well as the forest.

## The highlights of memory-relevant events for searching memory cells

 The mechanism underlying learning and memory has been systemically studied for more than one century ^6, 34– 36^. Many observations and concepts have been proved to be solid, however, data inconsistencies and indication controversy still block our clear vision to abstract cellular architectures and molecular profiles. Major reasons for this vagueness may be due to lack of the reliable standards to uncover memory cells in neural circuits (the basic units of memory trace or engram) that encode specific signals stored, to identify molecules resided in memory cells specifically for their recruitment, as well as to validate behaviors specifically initiated by memory cells. In order to set up reliable criteria for judging whether memory cell ensembles or memory traces being recruited are correlated to memory formation and retrievals, the changes at the levels of molecules, neurons and behaviors in learning and memory should be precisely estimated.

 In the study of memory retrieval, the stimulus-induced or cue-induced expression of specific behaviors that have been presented during learning events and memory retrievals are better used to denote the persistent presence of memory traces. The use of this strategy may have the following shortfalls. Behaviors, perceptions and cognitions are quickly developed postnatally ^37, 38^. Postnatal developments in perception and cognition versus behavior are not parallel in aspects of their patterns and contents ^39– 41^. The number of arm/body language patterns is much lower than the number of memory contents as well as the number of verbal language patterns. Although the number of memory contents is matched with the number of verbal language patterns, one arm/body language pattern may represent several memory contents. For instance, the thumb-up gesture usually represents memory contents relevant to all positive events. Moreover, patterns and varieties in sensory input signals, memory contents, cognitive processes or emotional reactions are much enriched in comparison with behavioral patterns that are presented by common output pathways, i.e., all of these signals, contents and processes are expressed by behavioral output patterns and pathways in the limit number. For instance, the “OK” gesture is used to express appropriate sensory stimulations, good perception, successful memory retrieval and other good cognitions. In other words, behaviors may not well present the retrievals of specific memory contents, except for verbal language. This limitation of behavioral presentation to memory and cognition may be an issue in the study of memory retrieval based on behaviors in animals. For instance, body freeze and involuntary/voluntary shaking used to signify fear memory can be induced by extreme fear, anxiety, emotional reactions (e.g., anger and fighting) and physiological processes (e.g., hypothermia and hypoglycemia). Furthermore, the brain in matured human beings and animals is highly wired, and its different regions are interconnected ^42^. Stimulations to potential memory traces by electrical, optogenetic or chemogenetic approaches being given to a location of the brain may indirectly activate other areas connected with this location to induce memory-related behaviors indirectly or behaviors across or similarly to memory retrieval, i.e., the replay of “memory-related behaviors” may not be directly or realistically controlled by memory traces.

The learning process generally includes the associative acquisition of simple signals or unitary signals and the reorganized acquisition of these unitary signals. At young age, language learning includes letters and words, and knowledge learning is mainly definition and concept. After the primary learning of unitary signals, the advanced learning moves forward to more complicated concepts by the reorganization of unitary signals, i.e., the acquisition of sentences and articles by the association of letters and words in language, as well as the acquisition of principles and theories by the association of definition and concepts in knowledge. Unlike verbal presentation, arm/body behaviors are not obviously upregulated to the complicated version for expressing the advanced language and knowledge, such that similar behaviors in different postnatal periods may represent different contents and knowledge. In other words, memory retrievals represented by similar arm/body behaviors likely include different contents. Taken together, we assume that the retrievals of stored signals by the replay of similar behaviors may be changeable in their contents spatially and temporally, i.e., behavior replays are unreliable, except for the reoccurrence of cues-induced behaviors.

 At the cellular level, the basic units in the brain are neurons and glia cells. It is important to figure out new features of those neurons that have been recruited as memory cells for storing specific signals, in order to map their working principles during memory formation and retrieval. In addition to their conventional natures, such as innate synapse input, synapse transmission, neuron excitability and excitation outputs, memory cells theoretically encode the newly learned signals and receive new synapse inputs that carry these newly learned signals. As the most common style of learning and memory is associative in nature, i.e., the integrative storage of associated signals, associative memory cells recruited should encode both innate signals and new signals, as well as receive new axon projection and synapse innervations in addition to the innate input. In this regard, the detection of new synapse innervations and multiple signal encoding by recording approaches (cell electrophysiology and imaging) is critically important in reporting the finding of memory traces. Moreover, learning and memory involve the memory of unitary signals in the young and the memory of complicated signals, i.e., reorganized unitary signals, in the later period of development. Individual associative memory cells presumably encode multiple unitary signals, and their assemblies work together to store unitary signals in different reorganizations. In other words, ensembles of associative memory cells store advanced knowledge contents in specific spatial and temporal patterns ^7^.

 In the principle of cell physiology, neuronal excitation is driven by synapse inputs and neuronal excitability is controlled by spiking threshold ^43, 44^. The patterns and frequencies of neuronal spikes are influenced by synaptic transmission and spiking threshold, i.e., there is a proportional correlation between the intensity of neuronal activities and the strength of synapse inputs, but not the nature of input contents. Similarly, activity patterns and spiking frequencies of memory cells in memory traces can denote their activity strength but not memory contents, such that the replay of certain neuronal activity patterns, such as spontaneous sharp-wave ripple, indicates the reemergence of neuronal activity strength without the necessary implication of memory features and contents being encoded. Cues-induced neuronal activity may reflect the retrieval of memory contents. Learning-cues should be used to track the distribution of associative memory cells in the different grades of memory traces.

 In order to figure out the features of memory cells and their working principle during memory retrieval, we expect to reveal the featured molecules in associative memory cells to label these memory cells. Based on analyses above, the formation of memory cells recruited from neurons involves axon projection and synapse innervation, two nonspecific processes in neurons. In this regard, the elucidation of molecular markers for memory cells is challenge. Recently, immediate early genes have been used to label memory cells ^45, 46^, which depends on a proposal that activated memory cells express immediate early genes ^47, 48^. Unfortunately, the expression of immediate early genes is proportional to the strength of neuronal activities which are not specific for memory cells. In this regard, the neurons with combo features in the labeling of immediate early genes, the innervation of new synapses and the encoding of new/innate signals are better termed as engrams.

 In summary, neurons that meet all of the following criteria, such as cues-induced behaviors, cues-induced replay of neuronal activities, new synapse innervations and active molecule labeling can be defined as memory cells. Strategies to find out memory traces that meet these criteria are discussed below.

## Strategies used to search basic units of memory traces

Two issues are important to clearly address the cellular and molecular mechanisms underlying associative memory formation and retrieval, i.e., animal models and strategies for searching cell assemblies in memory traces or engrams. Based on the studies of learning and memory over centuries, we summarize the animal models and strategies that have been used.

As associative learning of multiple signals is the most common approach of signal acquisition in life, the mechanisms underlying the integrative storage of these associated signals should be addressed by using appropriate animal models featured by association. A few animal models have been used to the study of associative learning and memory, such as classical conditioning that includes Pavlov’s conditioned reflex, eyeblink conditioning and fear conditioning in rodents and withdraw reflex in Aplysia, as well as operant conditioning that includes various types of reward memory (e.g., operation plus reward and place plus reward) in mammalians ^45, 49– 63^. In these models, a stimulus is unconditioned, whereas another stimulus is conditioned. However, in human beings, the memory of associated signals occurs by the signal inducing the recall of its associated signals, or the other way around. This reciprocal retrieval of associated signals constitutes the basis of associative thinking, logical reasoning, computation and imagination in forward and backward manners. It seems to us that these animal conditioning models do not signify whether the air-puffing to the cornea or the electric shocks to the feet is able to induce the recall of sound signal after the onset of eyelid-blinking conditioning or fear conditioning. That is, these conditioning models may not be ideally used to study associative memory. Moreover, electrical shocks may activate the whole brain by spreading electrical current in the body, so that the association is not region-specific in the brain ^7, 8^. Compared with electrical stimulations used in the study of fear memory, physical and psychological stresses in social interactions are closer to real life situations ^64– 66^. Recently, an animal model has been introduced to study associative memory in that the association of whisker and olfactory stimulations in mice leads to odorant-induced whisker motion and whisker-induced olfactory responses, a typical example of reciprocal retrieval of associated signals ^9– 11, 15, 16, 67^.

In terms of strategies to study associative learning and memory, theoretical analyses and experimentation *in vivo* are used ^68– 72^. Theologists in the field of learning and memory focus on drawing potential units for information storage in the brain, such as memory traces, engrams and cell assemblies. Experimenters make efforts to figure out molecular substrates and cellular architectures for memory formation. In order to prove causal relationships between newly formed neuron substrates and memory behaviors, three criteria should be met. The emergence of new substrates and architectures is parallel to memory formation. The downregulation of newly formed substrates and architectures substantially reduces memory formation through the approaches of surgical ablations to brain tissues, pharmacological blockades to neuronal activities and genetic knockout/mutagenesis to molecules in nerve cells or synapses. The upregulation of these newly emerged substrates and architectures significantly facilitate memory formation through the approaches of pharmacological, electrical or optogenetic stimulations to nerve cells and gene overexpression in neurons and synapses ^7– 9, 73^.

In addition to the term “memory traces” for information storage coined by the ancient Greeks, theoretical terms “engram and ecphory” have been suggested by Richard Semon ^74^, a renowned theologist in the field of learning and memory. Engram and ecphory correspond to memory traces and memory retrievals, respectively ^71, 75^. In addition, his view on the relevance of memory retrievals says that the interaction between the stored engram and retrieval cues may generate new engrams. As long as an engram-awakening stimulus is similar to an original stimulus, this incomplete retrieval cue is sufficient to retrieve the stored engram. Awakening the originally stored engram may generate a new engram related to this event. The old retrieved engrams and new engrams become associated through contiguity to strengthen original memory. Moreover, the simultaneous retrieval of multiple engrams with similar contents and their subsequent associations, i.e., resonance among engrams, would provide the basis for the complicated cognitive processes, such as abstraction, generalization and knowledge formation ^76^. This theory may be the first to hypothesize that awakening engrams is dynamic and use-dependent. Although the engram termed by Simon lacks experimental evidence during that period, his frameworks about engrams are consistent with the features of memory activities. For instance, more representations lead to deeper memory, and the repeated simultaneous recalls of similar memory contents induce them to be summarized, conceptualized and generalized. In brief, his work has led to the consensus of memory traces or engrams as the basis of information storage.

Donald Hebb, another well-known theologist, describes memory traces or engrams to be cell assemblies as the basis of memory behaviors. According to his and Penfield’s observation that the destruction of large amounts of cerebral cortices in human beings produces little effect on memory ^77, 78^, as well as Lashley’s experiments that the ablation of widespread cortices in animals does not induce parallel changes in memory behaviors ^79, 80^, he has proposed the term “cell assemblies” that are the widely distributed neural substrates for memory. Each cell ensemble is a group of interconnected cells in that their interconnections are formed during their simultaneous activities ^81, 82^. Since these cells are interconnected, the activity in this circuit is maintained briefly after the event, i.e., short-term memory. Activities recurring for a sufficient duration within this cell ensemble can induce growth or metabolic change that strengthens those interconnections among assembly cells, such that short-term memory is converted into longer-term memory ^82^. The strengthening of connections between presynaptic and postsynaptic nerve cells in their simultaneous activities confers these neurons the property of firing together and strengthening together, which has been hypothesized as being a neuron connection. The strengthening of neuron connections has been shown in long-term potentiation of synaptic transmission ^83, 84^. The high number of interconnections among cells may allow the entire ensemble to be activated if a subset of cells is activated by the process of pattern completion that induces memory retrieval. As Hebb’s cell assembly is widely distributed in and across brain areas, destruction in a small proportion of cells may not lead to catastrophic memory traces, or graceful network degradation, which may account for Lashley’s experimental results. In summary, Hebb’s theory has overlapped multiple spatial scales from the integrated synaptic strengthening (a microscale level) to cell assembly formation (a mesoscale level).

The computational simulation of neuronal substrates for learning and memory has been used to deliver the theoretical model of memory traces, in which the data for modeling is based on experimental results. In the study of neuronal and synaptic architectures for memory traces and memory related behaviors, there are clear indications that show the involvement of neuronal ensemble and synaptic plasticity in processes of learning and memory in spite of a lack of evidences about synapses, neurons and their plasticity specifically correlated to memory formation ^76, 85– 89^.

In summary, the study of memory formation by theoretical models has generated great frameworks that can provide useful guideline for addressing cellular mechanisms underlying learning and memory. However, these hypotheses about memory traces (or engrams) and cell assemblies have not indicated any insight about the integrative storage of associated signals and need be proved by experimentation. In experimental studies about learning and memory, three strategies can be used to confirm causal relationships between memory traces (cell assemblies) and memory-related behaviors. Memory traces should be detected during memory formation and cue-induced memory retrievals. The downregulation of memory cell assemblies can restrain memory-relevant behaviors. The upregulation of memory cell assemblies can facilitate memory-relevant behaviors ^7– 9, 73^. There are two usual methods to track memory traces (engram) or cell assemblies, i.e., the detection of memory cells during learning and memory and the activation of memory cells to retrieve memory-relevant behaviors. The detection of memory cells involves observing their responses to memory cues by electrophysiological recording and two-photon calcium imaging and localizing their distribution by AAV-carried fluorescent neural tracing after memory formation. The activation of memory cells can be done by electrical, pharmacological, optogenetic or chemogenetic stimulations to induce the emergence of memory-relevant behaviors ^7^. It is noteworthy that memory traces are widely distributed in the brain and brain areas are interconnected. These stimulations may lead to the antegrade and retrograde activation of neural pathways. The indirect activation of memory traces is unable to localize primary versus secondary allocations for memory formation.

Neuronal activities are indicated by electrical signals generated on the cell membrane and calcium signals raised in cells, such that the recording of electrical signals and the imaging of intracellular calcium dynamics can be applied to track cell assemblies relevant to memory formation and retrieval, i.e., the functional detection of memory traces ^90^. The electrophysiological recordings by electrodes or electrode array have been used to detect the replays of neurons in the hippocampus, visual/auditory cortices, the amygdala and ventral tegmental areas under different conditions, such as retrieval cues, wakefulness and sleep state ^30, 91– 104^. For example, coordinate interactions from the hippocampus to the prefrontal cortex and associative cortices, including parietal and midline areas but not primary areas, are involved in spatial memory tasks ^105, 106^. The cortical-hippocampal-cortical circuit is critical for memory consolidation ^107^. Hippocampal assemblies trigger neuronal activities in the ventral striatum during the replay of place-reward information ^55^. The acquisition of associative memory in the hippocampus initiates a gradual-to-stable encoding process in the medial prefrontal cortex without continued trainings ^29^. Emotional memory is reactivated in the hippocampus-amygdala system during the sleeping state ^108^. These data from this functional study are supported by anatomical evidence within the hippocampus, the prefrontal cortex and the thalamic nucleus ^109^.

Recently, two-photon cell calcium imaging *in vivo* ^110– 112^ has been used to detect memory traces or memory cell assemblies in cerebral cortices. For instance, the gradual emergence of neuronal activity relevant to spatial memory in the retrosplenial cortex, which is the major recipient of hippocampus, depends on the intact hippocampus. Indirect connections between the retrosplenial cortex and the hippocampus indicate hippocampal influence polysynaptically within the neocortex, i.e., widely distributed memory traces in the hippocampus and cerebral cortices ^113^. Repetitive motor learning induces the formation of dendritic spines *in vivo* ^114^. Associative memory cells developed in response to retrieval cues are detected in primary sensory cortices and the prefrontal cortex ^9, 11, 12, 32^. Thus, memory traces or cell assemblies can be tracked by electrophysiological and imaging recordings based on their activities in response to retrieval cues and during memory-relevant behaviors.

Importantly, the data above support the functional presence of memory traces or cell assemblies, which is better validated by morphological traces, i.e., their morphology and distribution should be quantified and localized. Two methods can be used for this purpose: the trace of their synapse innervations from axon inputs that are carrying the learned signals, as well as the labeling of these cell assemblies by molecules specifically relevant to memory. In the study of associative memory cells, fluorescent expression mediated by adeno-associated virus (AAV) vectors in neurons and their axons has been done by injecting AAVs, tagged with genes of fluorescent proteins, in the source side of predicted memory traces and by detecting axon terminals and their target on associative memory cells ^9, 13, 15^. These associative memory cells receive both innate and new synapse innervations. It is noteworthy that the combination of tracing new synapse contacts and labeling memory assemblies with memory-relevant molecules would be an ideal way to denote memory cell assemblies.

Neuronal activities may lead to a change in certain molecules ^115– 117^, so that learning and memory recruit the neurons to be memory cells presumably by molecular substrates. The labeling of memory cells by these molecules can be applied to indicate the allocation of memory cell assemblies, based on the facts that the stimulation of neurons couples with the expression of immediate early genes ^48^ and their expression in dendrites is regulated by synapse activity ^47^. For instance, immediate early gene Arc is specifically linked to the neural encoding process ^118^. Immediate early genes are widely expressed in the brain after fear memory and the number of labeled cells is positively correlated to fear memory behaviors ^45, 46^. It seems that there is an association between the expression of immediate early genes and the active strength of memory cells. The detection of immediate early gene expression is usually used to label the engrams, so that their morphologies and functions can be studied ^112, 119– 122^. However, the upregulated expression of immediate early genes is also associated with neuron hyperactivity, such as seizure discharge in epilepsy ^123– 126^ and neuron toxicity in the brain ischemia ^127– 129^. In these regards, immediate early genes may be suitable for identifying all of the neurons that are highly active. Genes and proteins specifically linked to memory cell assemblies and their memory contents remain to be explored ^68, 130^.

As the retrieval of memory-specific behavior is presumably based on memory traces that are formed during memory formation, the activation of memory cell assemblies to induce the emergence of memory-relevant behaviors should be included in the study of memory formation and retrievals. The use of this strategy is based on the positive correlation between memory cell assemblies and memory formation/retrieval, i.e., if some neurons are memory cells that store specific memory content, the activation of these cells by electrical, pharmacological and optogenetic methods should induce the representation of memory-relevant behaviors. Electrical stimulations to memory traces in the brain were given by Penfield who expected to localize the source of epilepsy ^131^. Stimulations to the temporal lobe in wakeful epileptic patients induced their memory recalls, i.e., engrams were detected in this cortical area ^132, 133^. Pharmacological stimulation has been used to activate the serotonin or norepinephrine system to examine the facilitation of memory formation by the transmitters successfully ^134, 135^. Recently, optogenetic stimulation has been used to activate memory engrams, which mediate fear memory and false memory ^22, 136– 139^. Therefore, these results support the positive correlation between memory traces and memory-related behaviors. It is noteworthy that the direct optogenetic activation of neurons without increases of synaptic strength and dendritic spine density leads to memory retrievals ^140^, implying nonspecific neuron activation. As pointed above, the wide distribution of memory traces in the brain and the interconnection among brain areas may result in the stimulations being antegrade and retrograde activations of neural pathways. The indirect activation of memory traces is unable to localize primary versus secondary allocations for memory formation.

Similarly, if behaviors related to specific memory content depend on memory traces that are formed during memory formation, the downregulation of certain molecules critical for memory cell assemblies by pharmacological blocking, gene knockout or optogenetic methods should prevent or attenuate the formation and emergence of memory-relevant behaviors, which is commonly used to address the causal relationships among molecular substrates, cellular architectures and memory formation. The first use of surgical ablation to search the distribution of memory traces or engrams was done by Lashley. Although he failed to localize memory traces, his studies imply the wide distribution of memory traces in the cerebral brain ^79, 80, 141^. The following studies indicate that the removal of the temporary lobe in human beings leads to the loss of recent memory due to the impairment of the hippocampus ^78, 142– 144^. In the study of memory traces using pharmacological reagents, recent memory can be blocked by using intracerebral injection of puromycin ^145, 146^. These studies reveal a causal relationship of memory traces in wide brain areas to memory formation and retrieval, although memory traces specific for content-related behaviors remain to be tracked and localized. With the advanced molecular biology, the downregulation of gene expressions by gene knockout ^147^ and optogenetics ^148, 149^ have been successfully used to find negative correlations among molecules, memory cells and behaviors. These studies provide strong evidence for the causal relationships among molecular substrates, cellular architectures and memory formation.

The advantages and disadvantages of these strategies and approaches have to be evaluated and validated. In logical analyses, parallel changes, negative correlations and positive correlations between functions and changeable factors should be met in order to ensure the causal relationship. Studies involving manipulations of molecules and cells causing changes to memory-relevant behaviors in these three criteria should be combinedly used to identify memory traces formed after learning. Consistent results are expected to reach the conclusion. However, inconsistent results may occur in these studies. For instance, silencing and stimulating the patriate cortex lead to inconsistent results in memory retrieval. Parietal lesions do not normally yield severe episodic-memory deficits, whereas parietal activations are seen frequently in the function-neuroimaging studies of episodic memory ^150^. These two categories of evidence suggest that the answer to the puzzle requires us to distinguish the contributions of dorsal versus ventral parietal regions and the influence of top-down versus bottom-up attention on memory. The features of memory traces or engrams based on these studies include the following. Memory traces encode the trained signals, receive synapse inputs and undergo synaptic plasticity ^35, 69– 71^. The activation of memory traces evokes strong memory retrieval. Memory events are upregulated by norepinephrine and serotonin. How memory traces memorize multiple associatively learned signals needs to be addressed by observing cells in memory traces that encode the associated signals.

## Associative memory cells as basic units of memory traces

Associative learning includes the acquisition of associated signals that are basic features of various objects, knowledge and experience as well as the acquisition of the complicated signals that are reorganized from those basic featured signals in intramodal or cross-modal manner. Associative memory stands for the integrative storage and the distinguishable retrieval of these associated signals in neurons. Associative memory cells are presumably basic units to fulfill these processes during associative learning and memory by encoding multiple associated signals as well as receiving innate and new synapse innervations in the cerebral brain ^7, 8^. The integrative ability of associative memory cells indicates that activity-dependent synaptic plasticity in a single neural pathway, such as long-term potentiation and depression of synaptic transmission ^83, 151, 152^ and activity-dependent neuronal plasticity ^43, 153– 155^, may not be directly involved in the integrative storage of multiple associated signals, though this plasticity may influence memory retrievals ^7, 8^.

In terms of the location of information storage, memory traces appear to be widely distributed in the brain, such as the hippocampus, amygdala, motor cortex, sensory cortices and associative cortices ^3, 12, 21, 22, 25, 26, 28, 30, 106, 156– 160^. Memory contents reside hypothetically in cell assemblies by the strengthening of neurons’ interconnection that is triggered by their correlated activity in information acquisition ^81^. These studies do not explain why cell assemblies are widely distributed and how plasticity at synapses and neurons coordinately integrate associated signals for their storage in primary and secondary manners, i.e., the characteristics and working principle of these neurons that coordinately encode associative memory ^7, 8^. Neuronal and synaptic plasticity cannot interpret memory patterns, e.g., explicit versus implicit memory, declarative versus non-declarative memory, episodic versus semantic memory and memory transformation among these patterns ^33^, the temporal features of associative memory as well as the contribution of associative memory to cognitive processes, e.g., associative thinking and logical reasoning. How endogenous signals generated in associative thinking and logical reasoning are memorized for future representation remains unknown. How memory is encoded under different consciousness states needs to be addressed. The natures of these cell assemblies, the patterns of their connection strengthening and the coordination of their encoding memory need to be examined in a comprehensive manner.

**Figure 1.**
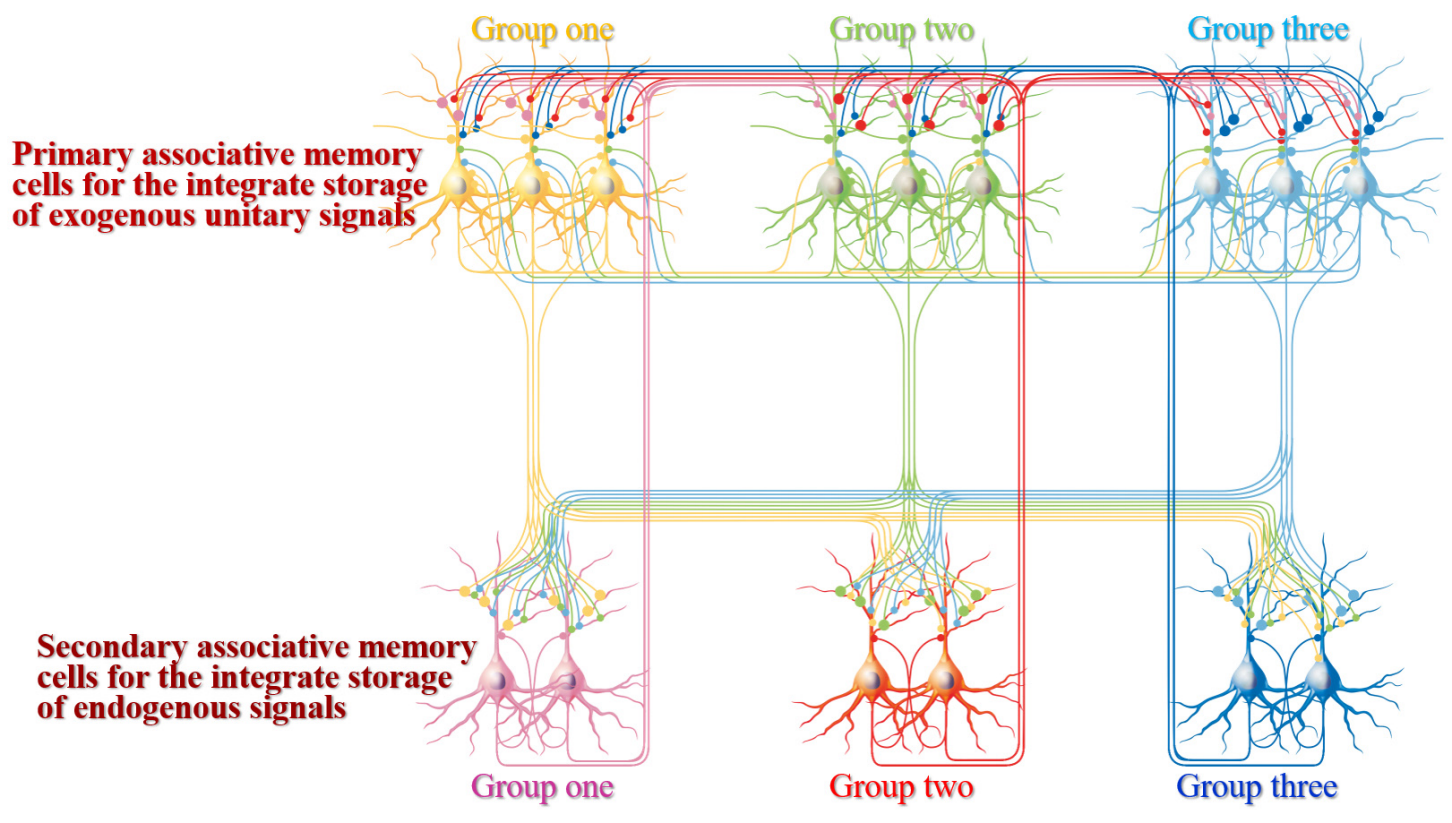
Associative memory cells and their connections. Three groups of primary associative memory cells (blue, green and yellow) in sensory cortices are synaptically innervated. Three groups of secondary associative memory cells (blue, red and pink) in brain areas relevant to cognition, emotion and behaviors are synaptically innervated. Mutual synapse innervations among associative memory cells in each group are intramodal, and mutual synapse innervations among three groups of associative memory cells are cross-modal. The axons of primary associative memory cells convergently and broadly innervate secondary associative memory cells, whose axons project back to primary associative memory cells. All neurons possess innate synapse innervations (yellow axons). The synapse innervations among the functional corresponding groups of primary and secondary associative memory cells are labeled by bigger presynaptic boutons.

Associative memory cells that encode multiple associated signals as well as receive innate and new synapse inputs have been detected to be recruited by the coactivation of cortical neurons ^7– 9, 11, 67^. The coactivation of sensory cortices evokes their mutual synapse innervations, and recruits associative memory cells to integrate and encode associative signals ^10, 11, 16^. Based on mutual innervations among associative memory cells ^9, 11, 15^, the associations of sensory signals for their integrative storage make each signal induce the recall of its associated signals in a reciprocal manner. In the meantime, these primary associative memory cells in the sensory cortices send their axonal projections toward brain areas relevant to cognitions, emotions and behaviors, and undergo synaptic convergence with individual neurons in these areas during logical reasoning and associative thinking to recruit them as secondary associative memory cells ^7, 8, 12^. In this regard, mutual synapse innervations among primary associative memory cells in sensory cortices and their innervations to secondary associative memory cells in brain areas related to cognition, emotion and behavior constitute the basic cellular architecture for the reciprocal recall of associated signals, the automatic conversion of associated signals during their recalls and cognition at the high orders ^7, 8^ ( Figure 1). In addition to the learning of associated signals from cross-modal sensory cortices, the acquisition of associated signals can be achieved in one of intramodal sensory cortices, such as the association of letters or words in the auditory cortex, the association of unitary images in the visual cortex, and so on. The recruitment and features of these associative memory cells are described below.

**Figure 2.**
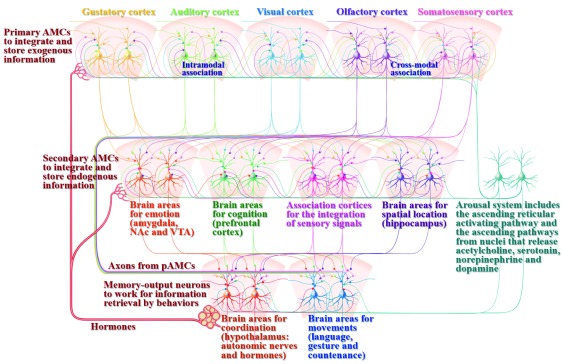
Associative memory cells and their working principles in the memory, cognition and emotion. Associative learning and memory include the acquisition of associated signals, the integration and storage of exogenous signals, the integration and storage of endogenous signals, as well as memory retrieval through behavioral presentation. Associative memory cells (AMCs) are classified into primary AMCs (pAMCs) in sensory cortices, including visual, auditory, olfactory, gustatory and somatosensory cortices for the integrative storage of exogenous associated signals, as well as secondary AMCs (sAMCs) in brain areas related to cognitive processes (logical reasoning, associative thinking, computation, imagination, concept, judgement conclusion, decision and so on in the prefrontal cortex), emotional reactions (fear, aversion, happiness, angry and so on in the amygdala, ventral tegmental area (VTA) and nucleus accumbens (NAc)), sensation integration (understanding and perception in association cortices) as well as spatial localization in the hippocampus. pAMCs are mutually connected through cross-modal and intramodal synapse innervations for the integrative storage and the reciprocal retrieval of associated signals. The axons of pAMCs convergently innervate onto sAMCs for cognition, emotion and spatial localization. sAMCs are mutually connected through their synapse innervations for the integration of cognition, emotion, perception, localization and so on. All of these primary and secondary associative memory cells will send their axons toward brain areas relevant to behaviors (language, gesture and countenance in motion cortices) and their coordination (the systems for maintaining internal environment, e.g., the hypothalamus to control autonomic nerves and hormones). Cross-modal associative memory cells are recruited by mutual innervations among sensory cortices or between cognition- and emotion-relevant brain areas. Intramodal associative memory cells are recruited by mutual innervations among the neurons in single-modality sensory cortex, cognition brain area or emotion brain area. In addition to the activation by innate input and new synapse innervation from the coactivated brain regions to integrate and encode associated signals, associative memory cells are activated by the arousal system, including the ascending reticular activating pathway in the brain stem and thalamus as well as the ascending activating pathways from the cholinergic nuclei, midbrain raphe nuclei, locus coeruleus and substance nigra that release acetylcholine (ACh), serotonin (5-HT), norepinephrine (NE) and dopamine (DA), respectively, which can maintain wakefulness, permit normal consciousness as well as grant specific alertness and attention. In addition, associative memory cells are regulated by hormones that are released from the hypothalamus-pituitary-glands. The upregulations of AMC number and activity strength can facilitate memory to be impressive, or vice versa. The function downregulation of motion-relevant brain regions leads to the inability of memory retrieval and presentation.

**Figure 3.**
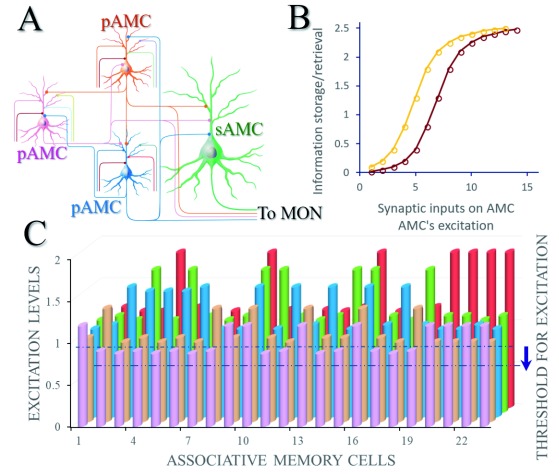
Associative memory cells and their functional states play the critical role in the integrative storage and the reciprocal retrieval of specific associated signals. **A**) A basic neural circuit (memory trace) for associative memory includes primary associative memory cells (pAMC) and secondary associative memory cells (sAMC). Each of these primary associative memory cells receives synapse innervations from the innate inputs (their colors correspondent to those of the cell bodies), the input from the arousal system (dark red) as well as the mutual synapse innervations among them (i.e., from other primary associative memory cells). These primary associative memory cells send their axons convergently to secondary associative memory cells (green) and make synapse innervations. All of these associative memory cells send their axons to memory output neurons (MON). **B**) The relationships between the excitation state of associative memory cells and the strength of memory formation/retrievals. The excitation state of such associative memory cells is influenced by the number and the function state of their synapse inputs and by their own excitability. If the excitability of associative memory cells rises, their relationship curve (dark red) shifts towards the left (yellow) and the efficiency of learning and memory increases. **C**) denotes the relationship between different associative memory cells and their excitation levels. If the threshold to fire spikes (excitation) decreases, the relative excitation levels of associative memory cells increase, as well as more neurons will be coactivated for the recruitment and refinement of associative memory cells in memory formation and retrieval.

*Associative memory cells recruited in sensory cortices:* Associative learning by paring whisker, odor and tail stimuli in mice leads to reciprocal responses induced by each of these signals, such as odorant-induced whisker motion, odorant-induced tail withdraw, tail-induced whisker motion, tail-induced olfaction response, whisker-induced olfaction response and whisker-induced tail withdraw ^9, 11, 13, 15^. Their barrel cortical neurons are able to encode new odor and tail signals alongside innate whisker signal as well as receive new synapse innervations from the piriform and S1-tail cortices besides innate inputs from the thalamus ^9, 17^. Their piriform cortical neurons encode new whisker signal and innate odor signal, as well as receive new synapse innervations from the barrel cortex alongside innate input from the olfactory bulb ^15^. In other words, a portion of the sensory cortical neurons in mice after associative learning become able to encode associated signals as well as receive new synapse inputs based on their mutual innervations alongside innate input, which are named as associative memory cells ^10, 11, 13, 16^. These associative memory cells have been assured to include glutamatergic neurons, GABAergic neurons and astrocytes ^9– 11, 13, 15– 17^. Thus, the coactivation or simultaneous activity of sensory cortices can trigger the new synaptogenesis for mutual synapse innervations and the recruitment of associative memory cells for the storage of associated signals. The association of cross-modal sensory signals may occur among all of sensory cortices, such as visual signal with auditory, olfactory, taste and somatosensory signals; auditory signal with visual, olfactory, taste and somatosensory signals; and so on, i.e., primary associative memory cells can be recruited in auditory, visual, olfactory, gustatory and somatosensory cortices by their mutual synapse innervations ^7, 8^ ( Figure 2 and Figure 3).

Associative memory cells recruited through the coactivation of sensory cortices are diversified in their encoding abilities and contents. Some cells encode all associated signals (full associative memory cells) and others encode two or more signals (incomplete associative memory cells), e.g., triple, two or one of odor, whisker and tail signals ^9^. If neurons are activated together and wired together, the coactivated strengths among these sensory cortical neurons may be different based on their variable excitability ^44^. Neurons that encode one signal are called new memory cells or innate memory cells ^9, 11^. The recruitment of diversified populations of associative memory cells in their encoding ability dissects complicated events, objects or images into simple unitary signals for their storage, future retrievals in different patterns and the reorganization of unitary signals in future associative learning ^7^. In addition, the repeated coactivations of these sensory cortical neurons can facilitate the recruitment of full associative memory cells from incomplete associative memory cells, as well as the formation of more *en passant* synapses among their mutual innervations, so that the number and the activity strength of associative memory cells are upregulated ^32^. The proportional relationship among associative memory efficiency, associative memory cells and their plasticity ^9, 11, 13, 14, 161, 162^ indicates an activity-dependent positive cycle between the recruitment and refinement of associative memory cells ^7^.

A feature of associative memory cells is the mutual axon projections and synapse innervations for encoding multiple associated signals. The molecules potentially responsible for axon growth and synapse formation are likely substrates underlying the recruitment of associative memory cells. Current studies indicate that antagomirs for microRNA-324 and microRNA-133a, through influencing *Ttbk1* and *Tet3* expression, attenuate associative memory, new synapse innervation and associative memory cell formation ^9, 73^. The downregulation of miRNA-342 expression and the upregulation of *Nlgn3* and *Nrxn1* expression are coupled with the recruitment and refinement of associative memory cells ^15, 17^. These genes and proteins are related to axon prolongation and synapse formation. Thus, the recruitment of synapse innervations and associative memory cells may be based on a chain reaction from intensive neuronal spikes to microRNA-regulated genes and proteins that specifically manage axon prolongation and synapse formation ^9, 13, 73^. In addition, the inhibition of sensory cortices blocks associative memory ^11, 13^ and the injection of microRNA antagomirs into sensory cortices lowers the strength of associative memory and the recruitment of new synapse innervations and associative memory cells ^9, 73^. Therefore, the primary location to encode associative memory is likely in the sensory cortices, where mutual synapse innervations and primary associative memory cells are recruited ^7, 8^.

The pair-encoding neurons that encode two signals, similar to the encoding property of associative memory cells, have been detected in the animal visual cortex *in vivo* ^2, 104^. These pair-encoding neurons in intramodal cortices may work for the integrative memory of the associated signals inputted from a single sensory modality, such as associated photon beams in images to the visual system, associated odor signals to the olfactory system, associated letters and words to the auditory system and so on ( Figure 2). It should be emphasized that the morphological evidence about mutual synapse innervations among the pair-encoding neurons in single modality cortices remains to be indicated.

As nerve cells, associative memory cells recruited in sensory cortices have specific features for associative memory and general features for neurons, in which specific features are used as criteria for identifying whether the neurons detected are associative memory cells. As their coactivation via the synchronous activity of cortical neurons triggers their mutual synapse innervations and recruits them as associative memory cells, the specific features of associative memory cells include the following ^7, 8^. Associative memory cells receive new synapse innervations from coactivated sensory cortical neurons for their mutual connections alongside innate sensory input. Associative memory cells encode new and innate associated signals for their integrative storage. Their axons convergently project to and synapse onto the neurons in brain areas relevant to cognitive processes, emotional reaction and behaviors. Their recruitment is controlled by microRNA-regulated genes and proteins that manage axon projections and synapse formations ^9, 13, 73^. The mutual synapse innervations among associative memory cells allow the reciprocal recall of associated signals and the conversion of signal retrieval among different modalities, e.g., image signals are presented through verbal language, verbal signals in stories are presented by visual diagrams. Their synapse convergences onto downstream neurons and the activation of associative memory cells permit logical reasoning, associative thinking, computing and so on. In general, for neuron and function outcome, the number and the function state of associative memory cells influence memory strength and maintenance. The number of associative memory cells is affected by their mutual synapse innervations under the induction of coactivation strength and repetitive coactivations as well as by developmental stages ^9, 11^. The functional state of associative memory cells is influenced by the strength of innate and new synapse inputs, their ability to convert synaptic analogue signals into digital spikes, as well as their ability to output spikes ^44, 163– 165^. In addition, glutamatergic associative memory cells will suppress the activity of other neurons through GABAergic associative memory cells and lateral inhibition to have themselves to be dominantly active for memory retrieval ^16, 17^.

In summary, synapse innervations to associative memory cells determine the specificity of memory content. The number and functional state of associative memory cells as well as the connection and activity strengths in their synapse inputs and axon output partners influence the power and persistence of memory and retrievals ^9, 14, 73, 162^. For instance, barrel cortical neurons receive new synapse innervations from the piriform cortex after associative learning alongside innate inputs from the thalamus. Synapse activities in the pathway of odor signal will drive barrel cortical neurons toward spiking threshold under the basal activity of thalamic inputs. Once the spiking threshold reaches, their spikes activate downstream motor cortical neurons for odorant-induced whisker motion. With these associative memory cells in sensory cortices ^9, 11, 73^, their axon-innervated downstream neurons are able to encode these associated signals ^12, 18, 23, 27, 29, 31, 166^. The stimulations to any of these areas in neural circuits from sensory cortices to behavior- and emotion-related brain nuclei induce memory representation ^21, 22, 25, 26, 28, 30^. It is noteworthy that there are around ten thousand types of proteins in living cells ^167^, which is much less than unit signals remembered in life, such as words, unitary images, odorants, and so on. As more than ten billion neurons reside in the central nervous system, those neurons with synapse interconnection, i.e., associative memory cells should be the basic units for memory traces, instead of the possibility of a specific protein for the given memory content.

*Associative memory cells in cognition-, emotion- and behavior-relevant brain areas:* In addition to primary associative memory cells in sensory cortices to integrate exogenous signals, secondary associative memory cells that integrate and store endogenous signals may be recruited in cognition, emotion and behaviors ^8^. The contents, processes and outcomes generated from logical reasoning and associative thinking can be remembered. Emotional reactions to various stimulations and operations can be recalled. All of these specific events in mind may be generated based on the associative storage of learnt exogenous signals in sensory cortices, such as images, stories, tastes and odors, and can be memorized in brain areas relevant to cognition, emotion or behaviors in the integrative manner for subsequent recalls. In terms of cellular substrates, the reorganized association of the stored signals in the sensory cortices may make primary associative memory cells to strengthen their mutual synapse innervations and convergent innervation on downstream neurons as well as to receive feedback synapse innervations during cognitive processes and emotional reactions. These downstream neurons become able to encode the associated signals and are recruited to be secondary associative memory cells that memorize specific contents generated in associative thinking and logical reasoning ^12, 32^. The feedforward and feedback interaction among primary and secondary associative memory cells make associative thinking and logical reasoning with the inclusion of sensory origins ^7, 8^ ( Figure 1 and Figure 2).

In terms of brain areas to produce secondary associative memory ^7^, prefrontal cortical neurons demonstrate a sustained activity after pair stimulations ^27, 29^. Cue-response neurons in the inferotemporal cortex are detected after associative learning ^23^. Neurons in response to conditioned and unconditioned stimulations and their response transformation are seen in the amygdala ^168^. Neurons in the hippocampus and amygdala are involved in contextual fear memory ^169^. Memory cell assemblies for temporal signals are overlapped and recorded in the hippocampus ^18^. The activation of engram cells in the amygdala or the hippocampus is sufficient to induce fear responses ^21, 22^. These data imply that memory cells are generated in the prefrontal cortex, hippocampus, amygdala and associative cortices for memory retrievals ^26, 170^. Whether these memory cells are synaptically innervated by primary associative memory cells in sensory cortices remains to be examined.

After associative learning by pairing whisker, odor and tail signals, neurons that encode three signals are detected in the motor cortex, prefrontal cortex and hippocampus ^12, 31, 32^, in addition to the barrel and piriform cortices ^9, 11, 15^. The responses of the neurons in the prefrontal cortex, the hippocampus and the motor cortex to the signals are attenuated by inhibiting barrel or piriform cortical functions. Their responses and plasticity are sustained in the barrel cortex for long-term and are decayed in the motor cortex after the pair training ends. Individual neurons in the prefrontal cortex, motor cortex and hippocampus receive synapse innervations from the coactivated sensory cortices after paired stimulations ^12, 31, 32^. These results provide functional and morphological evidences for the recruitment of secondary associative memory cells in the prefrontal cortex, the hippocampus and motor cortex through their coactivity with primary associative memory cells in sensory cortices ^8^.

Whether memory cells in the downstream of sensory cortices undergo cross-modal connections, similar to primary associative memory cells ^8^, appears indicated by recent studies. The pathway from the ventral hippocampus to the nucleus accumbens is involved in social memory ^24^. Engrams in the prefrontal cortex emerge after receiving inputs from the hippocampus and amygdala in contextual fear memory ^20^. Axon projection from the prefrontal cortex and hippocampus to the amygdala is formed during fear memory ^171^. The pathway from the prefrontal cortex to the striatum plays a crucial role in reward memory ^25^.

The characteristics of secondary associative memory cells in cognition- and emotion-related brain areas and association cortices are listed below. They receive new synapse innervations convergently from primary associative memory cells in coactivated sensory cortices in cognitive processes and emotion reaction. They encode endogenous associated signals from sensory cortices for the integrative storage. The association of cognition events and emotion reactions induces mutual synapse innervation among these secondary associative memory cells. Their axons project to memory-output cells in behavior-related brain areas for memory representation by language, countenance, gesture and writing. The number of secondary associative memory cells is influenced by mutual synapse innervation evoked by coactivation strength and repetitive coactivations during cognition as well as by development stage. The function state of secondary associative memory cells is influenced by synapse input, their ability to convert synaptic analogue signals into digital spikes and their ability to output spikes that drive memory-output cells. Synapse innervations to secondary associative memory cells determine the specificity of memory contents during cognitions and emotions. The number and excitability of secondary associative memory cells as well as their connection and activity strengths set up the persistence and power of memory formation and retrievals. Activations to secondary associative memory cells permit the rehearsal of associative thinking, logical reasoning and emotional reactions. It is pointed out that the outputs of secondary associative memory cells innervate brain areas, such as the hypothalamus and extrapyramidal system, to influence sympathetic/parasympathetic balance, temperature set-point, food ingestion and hormones to be involved in emotional reactions and behaviors.

Associative memory cells detected in cerebral cortices include glutamatergic neurons, GABAergic neurons and astrocytes ^9– 11, 13, 15– 17^. The connections between glutamatergic neurons and GABAergic neurons is mutually upregulated after memory formation ^15, 17^. These data indicate that all of these memory cells constitute the basic units to store specific associated signals. The activation of glutamatergic associative memory cells will cause them to be excited and their neighboring neurons to be inhibited by GABAergic associative memory cells and lateral inhibition, so that the memory of associated signals is maintained in a contrasting manner. In the meantime, these glutamatergic associative memory cells can limit themselves so not to become over-excited through GABAergic associative memory cells and recurrent inhibition ^7^. In terms of interactions among associative memory neurons and astrocytes, the working load of associative memory neurons can be supported by associative memory astrocytes, which transfer nutrients and waste products between neurons and blood vessels ^7, 9, 11^.

In addition to associative memory cells in cross-modal sensory cortices or among cognition- and emotion-related brain areas, associative memory cells can be located in intramodal cortices, such as associated photon beams in images to the visual system ^2, 104^, associated odor signals to the olfactory system, associated letters and words to the auditory system and so on ( Figure 2 and Figure 3). Neuronal afferent pathways for associated signals in a single sensory modality may innervate multiple groups of neurons, in which neurons in each group encode one of these associated signals. For instance, different groups of auditory cortical neurons receive neural afferents carrying different frequency sounds in a point-by-point manner and each group of neurons encodes one of specific frequency sounds. The different visual cortical neurons receive synapse innervations from different retina cone cells in a point-by-point manner. The coactivation of the neurons that encode different intramodal signals can induce their mutual synapse innervations, such that associative memory cells in a single modality of the sensory cortices are recruited. The associative memory cells in a given sensory cortex are recruited to memorize intramodal signals with different features, strengths and locations of input signals. With associative memory cells in intramodal sensory cortices, intramodal memory to associated signals is formed, e.g., image one induces image two recall, odor one induces odor two recall and word one induces word two recall, or the other way around ^7^. It is noteworthy that there is the time delay among intramodal signals, in which activity persistence in different sets of neurons in a given sensory cortex may grant the partially temporal overlap of their coactivity to recruit intramodal associative memory cells. The different proportions, activity strengths and connections of the intramodal associative memory cells are responsible for the storage and retrieval of intramodal signals with different features ^90^. Intramodal associative memory cells may also be recruited within one of brain areas relevant to cognitions and emotions ^8^.

In terms of the relationship between primary and secondary associative memory cells in memory traces and their role in memory-related processes, our proposed model is given as follows. Basic architectures for their working together include mutual synapse innervations among primary associative memory cells in the sensory cortices and their axon terminations onto secondary associative memory cells in brain areas relevant to cognitions, emotions and behaviors. Each set of primary associative memory cells connects one set of secondary associative memory cells reciprocally, whose functions are closely related ( Figure 1). The axons from all of these associative memory cells terminate to motor neurons for memory output (memory output cell) and innate reflex ( Figure 2). Mutual synapse innervations among primary associative memory cells constitute the interaction circuits for the reciprocal retrieval of associated signals by each of the sensory cues, as well as the automatic conversion retrieval of associated signals among different modalities ^9, 11, 15, 17^. The convergent synapse innervations from primary associative memory cells to secondary associative memory cells ( Figure 1) confer logical reasoning, associative thinking and other integrative cognition induced by one of cues ^12^. For instance, one of secondary associative memory cells is convergently innervated by three sets of primary associative memory cells that carry three kinds of signals, which maintain basic activities in this secondary associative memory cell. When an input cue activates three sets of primary associative memory cells by their mutual synapse innervations, these primary associative memory cells can convergently activate this secondary associative memory cell, in addition to its activation through the dominantly innate chain from one set of primary associative memory cells onto one set of secondary associative memory cells. In other words, three kinds of signals triggered by one of these cues drive this secondary associative memory cell to achieve the integration of three associated signals for associative thinking and logical reasoning. This integration is also facilitated by mutual synapse innervations among secondary associative cells that contribute to interactions of the higher order cognition and emotions. The divergent synapse innervations from primary associative memory cells to secondary associative memory cells ( Figure 1) mean that associated signals are stored in several brain areas for long-term maintenance with less chance of being lost, as well as being used for different cognitive processes and emotional reactions ^12^. In addition to this feedforward innervation from primary to secondary associative memory cells, there may be a feedback connection from secondary associative memory cells to primary associative memory cells, by which the learnt exogenous signals will automatically initiate cognition and emotions as well as endogenous signals from cognitive events and emotional reactions which usually contain sensory signal sources ^7^ and ( Figure 1 and Figure 2).

## The refinement of associative memory cells

Cell assemblies formed by connection strengthening through their correlated activities, especially the coincidence activity of presynaptic and postsynaptic cells, presumably work for learning and memory ^81^. This hypothesis is well matched by synaptic and neuronal plasticity ^69, 172– 174^, e.g., long-term potentiation and depression in synaptic transmission ^83, 152^ or neuronal activity ^27, 155^. Many studies about synapse and neuron plasticity were not carried out in memory cells despite brain areas presumably relevant to memory. Synaptic plasticity in a given neuronal pathway does not reveal how multiple signals are integrated and encoded in associative memory cells. These uncertainties raise the issue of how these data about plasticity are written in the profile of cellular mechanisms underlying associative memory. Based on current studies, there are two forms of plasticity in associative memory cells, i.e., the refinement during their recruitment for them to coordinate with each other and the refinement induced by cues to recall specific signals, both of which are activity-dependent based on coactivation among neurons ^9, 11, 13, 15, 17, 32, 73^, i.e., recruitment-related refinement and activity-dependent plasticity.

In the recruitment of associative memory cells from cortical neurons through their coactivation and mutual synapse innervations, the number of excitatory synapses and the transmission strength at each of these synapses on glutamatergic and GABAergic neurons are enhanced; the output of glutamatergic neurons is enhanced and the output of GABAergic neurons is weakened ^15, 17, 161, 162, 166^. In addition, the active intrinsic property of glutamatergic associative memory cells is upregulated and the excitability of GABAergic associative memory cells is downregulated ^15, 17, 161, 162^. Mutual synapse innervations among the associative memory cells are increased ^15, 17^. Increases in the driving force from excitatory synapses and in the excitability of memory cells, as well as decreases in the driving force from inhibitory synapses, shift the balance of these cortical neurons between excitation and inhibition towards excitation. Their high activity can attract more synapse innervations, recruit more glutamatergic/GABAergic associative memory cells, promote their functional state to an optimal level for information storage and facilitate the activation of these associative memory cells for retrieval of the associated signals ^11, 13, 14, 17^. The increased number and function of excitatory synapse inputs in associative memory cells strengthen the encoding ability and precision ^44, 164, 165^ for efficient memory formation and precise retrieval. If excitatory associative memory cells are over active, they can activate neighboring inhibitory neurons to prevent hyperactivity through recurrent negative feedback ^43, 44, 175^.

There are two forms of neuronal excitation plasticity to interpret how neuronal refinements are involved in the formation and the retrieval of associative memory, i.e., the downregulation of threshold potential to fire spikes and the upregulation of spiking ability to fire more sequential spikes. The intensive activity of cortical neurons by high frequency stimulus, similar to neuronal coactivation during associative learning, shifts spike threshold potential toward the resting membrane potential, so that the firing of neuronal spikes is facilitated ^155^. The intensive neuronal activity also upregulates the capacity to fire sequential spikes ^27, 69^. Both mechanisms elevate the neuronal capability to encode digital spikes, which strengthens a chain reaction from spikes to microRNA-regulated expression of genes and proteins that facilitate the recruitments of new synapse innervations and associative memory cells ^9, 11, 15, 17, 73^ as well as the retrieval of the associated signals ^176^. These changes have been detected in associative memory cells ^15, 17, 162^. Thus, plasticity in neuronal excitability may play one of central roles in learning and memory, which is reiterated by a current review ^177^.

In the study of memory traces or cell assemblies, synaptic potentiation has been detected at engram cells in slices of the prefrontal cortex, the hippocampus and the amygdala ^140^ and the excitation enhancement of B51 neurons is isolated from Aplysia ^178^. In the study, by using cues to sensory inputs *in vivo*, activity-dependent potentiation in response to associated signals was evoked at input pathways in the active group of primary and secondary associative memory cells, and activity-dependent conversion from silent into active neural pathways in response to associated signals was initiated in the inactive group of associative memory cells ^7, 12, 32^. This activity-dependent upregulation in response to associative signals in the given group of associative memory cells may allow them to become more excited than their neighboring neurons and to be highly sensitive to the excitatory driving force from sensory cues, such that more associative memory cells are recruited by their increased mutual synapse innervations in response to all associated signals ^15, 17^. Furthermore, activity-dependent potentiation in response to associated signals can be induced through homosynaptic and heterosynaptic pathways ^32^, which facilitates the reciprocal recall and logical reasoning of those associated signals. Activity-dependent potentiation at associative memory cells in response to the associated signals inputted through new synapses may be mechanistically caused by the enhancement of individual synapses and/or the conversion of inactive or silent synapses into functional synapses ^179, 180^, since new mutual synaptic innervations have been formed among these associative memory cells ^7, 9, 11, 12, 15, 17, 32, 73^. In terms of function impacts, activity-dependent potentiation at primary associative memory cells may facilitate the memory retrieval of exogenous associated signals. Activity-dependent potentiation at secondary associative memory cells facilitates the memory retrieval of endogenous signals generated during cognitive processes and emotional reaction. Thus, the spontaneous or cue-induced recalls of these signals are emerged for the rehearsal of cognitions and emotional pulses. Recruitment-related neural potentiation and activity-dependent neural potentiation are supported by the fact that the enhancement of neuronal excitability is multi-grade in nature ^155^.

Recruitments of primary associative memory cells in sensory cortices and of secondary associative memory cells in cognition/emotion-relevant brain areas endorse the specificity of the storage of associated signals ^8, 9, 11, 13, 15, 17, 73^. The number and function state of associative memory cells influence the strength and maintenance of specific memory as well as the efficiency of memory retrieval ^9, 13, 14, 73^. Structural and functional plasticity at subcellular compartments of associative memory cell influences whether they sensitively integrate associated signals, precisely memorize these signals and efficiently trigger their target neurons for memory retrievals ^15, 17^. The maintenance of activity-dependent refinement at associative memory cells supports the period for them to be sensitive to the cue for memory retrieval. It is emphasized that both recruitment and refinement of associative memory cells depend on their simultaneous activity ^9, 11, 13, 15, 17^. The activities of associative memory cells as central point comprise coactivity-dependent positive cycle in their recruitment and refinement, i.e., activity together, mutual innervation together and strengthening together. Highly active neurons while receiving associated signals are recruited as associative memory cells and are functionally upregulated. The upregulated population and function state of associative memory cells during repeated learning processes recruit more associative memory cells and upregulate their active state further ^7^. Activity-dependent positive cycle in the recruitment and refinement of associative memory cells, which is based on the function compatibility between neuronal partners ^163^, can interpret realistic practices under conditions of normal consciousness and well attention, i.e., the more learning times is, the more associative memory cell recruitment/refinement is, and the more impressive memory is. It should be pointed that associative memory cells fall into the active group of neurons in the brain, but active neurons labeled by non-specific immediate early genes may not be memory cells.

In terms of the functional states of primary and secondary associative memory cells influenced by synapse inputs, the number and strength of the inputted synapses are proportional to the excitation levels of these associative memory cells ^9, 14, 73, 161, 162^. The increase of synapse inputs that carry specific memory content and their upregulation from receiving repeated cues drive associative memory cells to become more excitable for the retrieval of this specific memory and the full recruitment of memory cells. The increased activity of synapse inputs from the arousal system boosts associative memory cells to become more excitable for the retrieval of memory contents stored in a nonspecific manner. Moreover, the increase of excitability or the decrease of spiking threshold in associative memory cells will make them be easily activated for the retrieval of memory contents nonspecifically and the recruitment of more associative memory cells ^15, 17^. A theoretical illustration of associative memory cells driven by synapses and neuronal excitability is given in Figure 3.

The neurons in the central nervous system that are dominantly recruited as associative memory cells needs to be determined. Based on the principle that the simultaneous coactivation of cortical neurons and the activity-dependent positive cycle between the recruitment and refinement of associative memory cells are the primary driving force for the neurons being recruited as associative memory cells ^7^, we assume that the neurons with high levels of excitation and synapse inputs are preferentially are recruited as associative memory cells. In other words, the cortical neurons, which possess a lower spiking threshold caused by their activities, as well as stronger synapse inputs driven by attention calls from previously learned relevant associated signals carried by the synapses formed in those events or by the consciousness levels maintained by the arousal system plus memory, are favorably recruited as associative memory cells. These dominantly active neurons are always recruited to be associative memory cells at the first grade, their activation and recruitment trigger the neighboring neurons through their synapse connections to be more active and become associative memory cells in the second grade, and so on. This preferential grading of the recruitment of associative memory cells leads to a time sequence for groups of cortical neurons to be recruited as associative memory cells when multiple associated signals are exposed to learners sequentially, such as words by words in sentences or articles and images by images in visual or video views ^7^.

There are a few interesting observations about the recruitment and refinement of associative memory cells. The establishment of associative memory follows a development change, i.e., memory formation shows initial increase and then decrease with aging ^11^. Synapse and neuron plasticity mature during postnatal development ^155, 180^. These studies indicate the dominant roles of recruitment versus refinement of associative memory cells in memory formation and retrieval in different developmental stages. The activity-dependent recruitment of associative memory cells may play the dominant role in associative memory during early and young age, while the activity-dependent refinement of associative memory cells works dominantly after these stages. The knowledge learned in young age is in the form of relatively simple unitary signals whereas the knowledge learned in matured age is complicated in the form of reorganized unitary signals. In this regard, associative memory cells recruited in young age store unitary signals, and associative memory cells refined in matured age work by learning the reorganized unitary signals ^7^.

## Associative memory cells are modulated by transmitters and hormones

In addition to new and innate synapse innervations on primary associative memory cells, their convergent and divergent innervations on secondary associative memory cells and reciprocal synapse innervations among them ( Figure 1), such associative memory cells may receive synaptic innervations from the arousal system, including the ascending reticular activating pathway ^181, 182^ and the ascending activating pathway from the neuronal axons of the locus coeruleus, the midbrain raphe nuclei, the cholinergic nuclei and substance nigra ^183– 186^. The arousal system widely innervates the neurons in cerebral brain to maintain wakefulness and to permit consciousness through their released acetylcholine, serotonin, norepinephrine and dopamine. It has been proposed that this arousal system, under the conditions of alter and rewards, supports the coactivation of cortical neurons for their recruitment to be associative memory cells, as well as maintains the basal activity of primary and secondary associative memory cells ^7^ ( Figure 2). This proposal is supported by recent studies that memory formation and retrievals are upregulated by acetylcholine, norepinephrine, serotonin and dopamine ^134, 135, 187– 193^, although these studies are not focused on associative memory cells. In addition, there is a coordinated strengthening effect of serotonin and norepinephrine on associative memory cells to raise the efficiency of associative learning and memory ^32^. Serotonin increases neuron responses to synaptic inputs ^194, 195^, and dopaminergic neurons enhances synaptic bouton formation ^196^, indicating that these neurotransmitters act on synapses and neurons to facilitate memory formation.

In addition to neurotransmitters, hormones may influence the recruitment and refinement of associative memory cells. It has been found that estrogen upregulates the dendritic spines on hippocampal neurons ^197– 199^, luteinizing hormone downregulates cognitive processes and spine density ^200^, and gonadotropin-releasing hormone regulates spine density ^201^. Moreover, estrogen and luteinizing hormone can upregulate associative learning, but this upregulation is attenuated by their combined applications ^202^. These data indicate that monoamine transmitters and hormones modulate learning and memory. The targets of these molecules on memory cells need to be addressed.

## Associative memory cells in physiology and psychology

Associative memory cells are essential for memory formation, memory retrieval, cognitions and emotional reactions ^9, 11, 12, 15, 31, 73, 161, 162^. The features and activity principles of associative memory cells can be applied to construct a working map ( Figure 1 and Figure 3) relevant to associative memory by cross-modal or intramodal manners, which includes the efficiency of associative learning, the integrative storage of multiple signals, the strength and preservation of associative memory, the efficiency of memory retrieval, the transformation of simple to complex information storage, the temporal sequence of learning and memory to multiple signals, the correlation of associating memory to cognitive process and emotional reactions, and so on. The features and working principles of associative memory cells also assist with interpreting memory patterns, e.g., declarative (explicit) versus nondeclarative memory (implicit), episodic versus semantic memory and transformation between such patterns under the conditions of consciousness and attentions.

The simultaneous activity of the neurons among different brain areas is essential for recruiting new synapse innervations and associative memory cells. The coactivity of sensory cortical neurons by cross-modal or intramodal manners induces their mutual synapse innervations, so that these neurons become able to encode multiple associated signals, i.e., these neurons are recruited as associative memory cells ^9, 11, 15, 17^. The coactivation of these primary associative memory cells also drives their axon prolongation and convergent synapse innervations onto the neurons in cognition and/or emotion-relevant brain areas, recruiting them as secondary associative memory cells in logical reasoning and associative thinking ^7, 12, 31, 32, 166^. These associative memory cells based on their synapse inputs and mutual synapse innervations constitute memories specific to associated signals. The activity-dependent positive cycle in the recruitment and refinement of associative memory cells recruits more associative memory cells to enhance memory strength and maintenance ^7^. These data provide new insights for memory formation, suggesting that mutual synapse innervations among primary associative memory cells endorse a reciprocal retrieval of associated signals and that secondary associative memory cells based on synapse convergences from primary associative memory cells function in associative thinking and logical reasoning. These results in activity together, connection together and strengthening together also upgrade a hypothesis by Hebb that the repeated coactivation of interconnected cells evokes the strengthening of neural wire to form cell assemblies for memory ^81^.

Associative memory formed by the association of multiple signals from cross-modal sensory modalities is commonly seen in life, such as the association of visual and auditory signals. Memory retrievals can be achieved by the automatic conversion of visual signals into verbal signals, or other way around, in addition to the retrieval reciprocally induced by either of the associated signals ^7^. For instance, images in movies or videos can be recalled and represented by verbal styles. The contents in verbal stories can be recalled as diagrams. Primary associative memory cells, by mutual synapse innervations among cross-modal sensory cortices, may contribute to the reciprocally induced retrieval and the automatic conversional retrieval of associated signals among cross-modal sensory modalities. Similarly, the intramodal association of multiple signals is commonly seen, such as different objects in single view and different words in single sentence. Primary associative memory cells by mutual synapse innervations ^7^ and pair-encoding ^2, 104^ in the single sensory cortex endorse memory retrieval in a picture-by-picture or word-by-word manner. It is noteworthy that signals in the visual system and the auditory system are usually complicated. An image consists of numerous photon beams with various light strengths and colors. Each sentence consists of many words and letters. Physiologically, the images that consist of numerous photon beams with different spatial distributions and light strengths are detected by different cone cells in the retina, which transmit these photon signals through visual nerves to visual cortical neurons in a point-by-point manner. Sound wave frequencies from words and letters are detected by hair cells in different segments on the cochlea base membrane, where hair cells are stimulated and their electrical signals are transmitted via auditory nerves to auditory cortical neurons in a point-by-point manner ^203^. How these unitary signals included in an image or a sentence are reintegrated and memorized in cerebral cortices is largely unknown ^7^.

In line with the principle of activity together, connection together and strengthening together ^7^, the coactivations of auditory cortical neurons, which receive synapse inputs from hair cells on cochlea base membrane and encode words or letters with different sound frequencies in early life, induce mutual synapse innervations among these neurons to recruit intramodal primary associative memory cells that store these unitary sound signals. As cortical neurons possess a few folds of differences in their excitability ^44, 204^, it may be postulated that the neurons with the highest excitatory state are dominantly activated. The afterdischarge of the neurons initially activated by the first letter or word coincides with the discharge of the neurons activated by the second ones, the afterdischarge of the neurons for the second letters or words coincides with the discharge of neurons for the third ones, and so on. The coactivation of these neurons evokes their mutual synapse innervations, which may constitute the integrative storage of letters in a given word or words in a given sentence. In repeated learning of this sentence or word, this group of auditory cortical associative memory cells is strengthened in their mutual synapse innervations and activities. The recruitment and refinement in this group of auditory cortical associative memory cells confer the consolidated memory of this word or sentence for subsequent retrievals. In the subsequent lifespan, sound signals to the auditory system become complicated, which are often the reorganization of unitary sound signals including letters and words. The learning of these reorganized unitary signals will strengthen their correspondent associative memory cells that have stored unitary sound signals via their synapse innervations and excitability, in order to encode these newly listened words and sentences, which will be preferentially activated in memory retrievals. In addition, glutamatergic associative memory cells suppress the activity of other neurons through GABAergic associative memory cells and lateral inhibition to have themselves activated preferentially for memory retrievals ^16, 17^.

Similarly, the coactivation of visual cortical neurons that receive point-by-point synapse innervations from retina cone cells in early life evokes mutual synapse innervations among these neurons in order to recruit intramodal associative memory cells that store unitary signals (photon beams with different intensity and color) in visual images. There is a proportional relationship between neural activity strength and stimulus intensity ^17^, so the neurons receiving the stronger light are more active. In line with the principle of neurons becoming active together, connecting together and strengthening together ^7^, mutual synapse innervations among these strongly active neurons will be dominant. These active neurons are recruited to become a group of intramodal primary associative memory cells to fulfill the integrative storage of strong light beams in given visual images. In the meantime, the axons of these associative memory cells may project to visual association cortices ^205, 206^ and make convergent synapse innervation onto their neurons to recruit secondary associative memory cells ^32^. This process of transferring from primary to secondary associative memory cells fulfills a transferring of image signals, especially strong photon beams, into the integrative storage at the secondary level as well as allows primary associative memory cells in the visual cortex to be able to receive new signals. As the neurons in the visual cortex correspond to the retina cone cells in a point-by-point manner, intramodal primary associative memory cells in the visual cortex receive major and minor synapse innervations based on their activity strength stimulated by signals from cone cells. Secondary associative memory cells in visual association cortices mainly receive convergent synapse innervations from active primary associative memory cells with major synapse innervations and active synapses converted from silent ones, such that major features in images are the integrative storage and subsequent retrieval. Our suggestions are supported by a recent report that visual association areas are recruited during memory formation ^207^. In subsequent associative learning, based on the reorganization of unitary signals in various new images, the portion of associative memory cells reactivated by those reorganized unitary signals will be integrated together through the conversion of inactive/silent synapses into active synapses among them to fulfill the integrative storage of new associated signals ^180^.

In practice, intramodal and cross-modal associative learning and memory occur simultaneously, especially the association of visual and auditory signals. For instance, unitary signals in visual images are associated to verbal signals during social activities, such as family activities, personal communications and classroom studies, in which each feature of a visual image is given clear definition by words or sentences. During social interactions, numerous associations are formed between unitary signals from the visual modality and words/phrases from the auditory modality. These associations at the unitary level will confer the learning of complicated information based on the reorganization of these unitary signals and the reorganized integration of associative memory cells. After cross-modal associative learning, individuals are able to fulfill the reciprocal recall of the associated signals, i.e., a signal evokes the recall of its associated signals, or other way around, as well as the automatic recall of signals through one modality that have been learned through another modality, i.e., the view of images is converted into verbal recall, or other way around ^7^. There are two mechanisms underlying these processes. In early life, the learning of associated signals from two or more modalities coactivates sensory cortical neurons in these modalities and induces mutual synapse innervations among them. Visual cortical neurons that encode unitary signals in the image mutually innervate with auditory cortical neurons that encode words or phrases, e.g., the neurons for unitary signals in an image “lemon” connect the neurons that encode words “lemon”, “yellow” and “oval”, based on their coactivation in initial learning ^7^. Numerous associations between unitary visual signals and auditory signals in social activities induce mutual synapse innervations between visual and auditory cortical neurons to form thousands and thousands of cell-pairs, primary cross-modal associative memory cells. Their active states grant memory retrievals. The accumulation of these associative memory cells that encode pairs of unitary signals will confer the learning of complicated signals based on the reorganization of these unitary signals. In postnatal development, the capabilities of axon growth and synapse formation are gradually attenuated ^11^. The learning of complicated signals during aging may utilize another mechanism for memory, i.e., the coactivity-dependent upregulation of associative memory cells in their excitability and mutually innervated synapses ^7, 17, 32^ or the activity-dependent conversion of inactive synapses into active synapses ^180^. As long as their function upregulations are maintained at a sufficient high level, these complicated signals can be retrieved automatically and/or by cues.

Through the coactivation-induced mutual synapse innervation for recruiting associative memory cells and coactivation-induced functional upregulation among associative memory cells, individuals can gradually memorize associated signals from unitary to reorganized unitary, i.e., the transformation of simple to complicated information storage, in a topic-related manner ^8^. Initially, the associations of simple images in the different intramodal features with words based on letters activate visual and auditory cortical neurons, respectively. With their mutual synapse innervation, intramodal and cross-modal associative memory cells are recruited including AMCs for pictures, letters as well as for picture and word. The repeated activations of these associative memory cells through practices will induce their activity-dependent plasticity and recruit more associative memory cells, i.e., coactivity-dependent positive cycle in the recruitment and refinement of associative memory cell. The first grade of associative memory cells is formed ^7^. With the accumulation of associative memory cells to store unitary signals, they become recruitment-ready neurons to be associative memory cells that encode complicated associative signals. The complicated visual and auditory signals can be associatively learned through activating the first grade of associative memory cells in visual and auditory cortices. Their mutual synapse innervation and activity upregulation lead to the formation of the second grade of associative memory cells that encode complicated images and sentences organized from unitary signals. Thus, numerous groups of the first and second grades of associative memory cells are accumulatively recruited in lifespan learning. In advanced learning, multiple grades of associative memory cells are recruited to encode more complicated signals. When the different groups and grades of associative memory cells are accumulated, subsequent learning may be based on their activity-dependent function upregulation, which makes them to be easily activated for quickly memory. Reading book or looking images induces the intensive activities in some groups of associative memory cells that encode these sentences and images, which leads to activity-dependent function upregulation at these associative memory cells. Their low threshold potential to fire spikes and active synapse inputs to drive these associative memory cells permit the cues dominantly to reactivate them for the recalls of images and sentences, and even the spontaneous activation of these cells to drive secondary associative memory cells for free associative thinking. The activity of these associative memory cells will lead to memory presentation by behaviors if they successfully drive the activation of memory-presentation neurons in the motor cortex ^7^.

It is noteworthy that the complicated signals can also be dissected and memorized through the formation of associative memory cells that are able to encode multiple signals ^9^. The complicated signals are composed of numerous unitary signals, which can be detected through the dissections by different sensory systems and intramodal sensory neurons. While learning these complicated signals, associative memory cells are recruited to integrate multiple simple signals, based on the random association of these unitary signals to induce mutual synapse innervations among their correspondent recruitment-ready neurons. These associative memory cells with different integrative ability to associated signals are recruited and the activation of portions of these associative memory cells leads to the selective recall of these complicated signals ^7^.

There are three resources of synaptic inputs to drive and maintain the activities of associative memory cells, including new synaptic innervations from coactivated brain areas, innate synaptic inputs formed during development, and synaptic inputs from the arousal system. The latter two synapse-driving forces activate the neurons ready to be recruited as new associative memory cells. The ascending reticular activating pathway from the brain stem and the thalamus receives various sensory inputs and widely innervates the entire cerebral brain to permit the wakefulness and consciousness ^181, 182, 208^. The ascending pathways from neuronal axons in the cholinergic nuclei, midbrain raphe nuclei and locus coeruleus innervate the forebrain to keep alertness and consciousness by releasing acetylcholine, serotonin and norepinephrine ^183, 184, 186^. This arousal system maintains the basal activity of associative memory cells, and confers them to integrate innate and new synaptic inputs specifically and to memorize associated signals. This arousal system may also activate recruitment-ready neurons to influence the efficiencies of associative learning, of associative memory cells to facilitate memory retrievals as well as of primary and secondary associative memory cells to permit the association of memory with cognitive process and emotional reactions.

Learning efficiency is influenced by neuronal excitability, synapse responsiveness and neurons ready to be recruited ^7^. Neurons ready to be recruited for storing new associated signals may be those cells that have been able to encode the storage of previous learnt signals from specific synapse innervations. These stored signals may be closely relevant to those associated signals that will be learned, and these ready recruited neurons can be activated by giving topic cues in the attention call. The number of the ready recruited neurons influences how the information is acquired and memorized easily as well as how the complicated signals can be efficiently learnt. That is a reason why the efficiency of associative learning is influenced by a fact whether individuals are knowledgeable in the topic to be learnt. In addition, the cortical neurons are diversified in their synapse inputs and intrinsic property ^44^, and the neurons with more synapse inputs and lower threshold potential are easily activated to fire spikes for high learning efficiency ^11, 162^, which triggers the chain reaction of intensive spikes and microRNA expression changes for axon prolongation and synapse innervations ^9, 73^. Thus, activity-dependent upregulations in neuron excitability and synapse innervations facilitate the recruitment of associative memory cells to influence learning efficiency.

The efficiency of memory retrievals is influenced by the number and function state of associative memory cells as well as the coactivity-dependent positive cycle between recruitments and refinements of associative memory cells ^7^. Under the conditions of normal consciousness and alertness, the recruited number of associative memory cells is positively proportional to the activated associative memory cells in memory retrieval, so that the efficiency of memory retrieval would be consistent to the efficiency of associative learning ^11, 162^, the functional state of associative memory cells affects how they are easily activated in memory retrievals ^17^, and the coactivity-dependent positive cycle between the recruitment and refinement of associative memory cells will add more associative memory cells into memory traces. Therefore, the efficiency of memory retrieval would be high under the conditions of normal consciousness and alertness. Whether the stored information can be successfully retrieved also depends on the function state of memory-output cells, since the function downregulation of memory execution cells in the motor cortex leads to the inability of memory retrieval (i.e., memory extinction) though primary associative memory cells are well-maintained in the normal function ^14, 31^. Thus, the high number and the active intrinsic property of associative memory cells in memory traces as well as the coactivity-dependent positive cycle of their recruitment and refinement lead to automatic memory retrieval after repeated learning and thinking without the need of cues.

In the transformation from exogenous signals to endogenous signals and their integrative memories ^7, 12, 31, 32, 166^, the efficiency to correlate associative memory with cognitive processes and emotional reactions is a critical issue. In this process, the interactions between primary and secondary associative memory cells by their mutual synapse innervations ( Figure 1) as well as the number and functional state of these associative memory cells should be taken into account during logical reasoning and associative thinking ^7^. Thus, cellular processes involved in the efficiency for the learning, storage and retrieval of exogenous associated signals may similarly work for the transformation of exogenous-to-endogenous signals.

In terms of the relationships between associative memory cells and memory patterns, such as declarative or explicit memory versus nondeclarative or implicit memory, episodic memory versus semantic memory as well as the transformation between these patterns, our interpretations are below. In spite of these psychological classifications, there is no clear border line to separate them. Declarative memory is an intentional remember with clear state under consciousness, while nondeclarative memory is an effortless remember with no conscious awareness ^6, 33^. In fact, implicit memory is formed in individuals by paying attention when they initially learn these processes and operations. With long-term practice to be skilled, the expression of such processes and operations are not necessarily to be fulfilled with conscious effort. Based on coactivity-dependent positive cycle in the recruitment and refinement of associative memory cells, the repeated coactivations of primary and secondary associative memory cells can recruit more associative memory cells and upregulate their function state ^7, 11, 15, 17, 32, 166^, as well as strengthen synapse connections from associative memory cells to memory-output cells in the motor cortex ^14, 31^, so that explicit memory can be converted into implicit memory. In other words, there may be the reverse relationship between the number and upregulation of associative memory cells and the requirement of consciousness, a homeostasis for memory retrieval. Implicit memory based on more associative memory cells that are easily activated is supported by phenomena that it can usually be expressed spontaneously. In explicit memory, episodic memory in individual events can be converted into semantic memory after the repeated associative thinking and logical reasoning strengthen associative memory cells that have stored a common signal of the events through central synapse innervations or place associative memory cells that have stored those events with similar topics together to reorganize them into a group of memory cells for the general concepts and to convergently innervate on another grade of associative memory cells in an abstraction manner ^7^.

Consciousness is the combinational state of wakefulness and memory for individuals to aware and identify themselves and objects in the environment ^209^. The normal consciousness may be based on the basal activation of associative memory cells by the arousal system and the specific activation of associative memory cells from their associated inputs triggered by sensory cues ^7^. Thus, the number and functional state of associative memory cells are proportional to the state of consciousness. The combination of consciousness and a specific alert constitutes the attention, in which a specific group of associative memory cells is activated for memory retrieval as well as the alert-relevant recruitment-ready neurons are coactivated for learning alert-relevant signals. Once individuals are under consciousness, they have two forms of logical reasoning and associative thinking, i.e., critical versus creative. The critical thinking activates more recruited secondary associative memory cells for the evaluation, while creative thinking may generate newer secondary associative memory cells for inspiration ^7^.

The awareness state can be classified into consciousness and unconsciousness. The sleeping can be fell into unconsciousness (slow wave sleeping) and incomplete consciousness (fast wave sleeping) ^209^. How do different groups of associative memory cells work together during fast wave sleeping or dreaming? Dreams are often accompanied by highly activities in electronic encephalograph and behaviors, such as rapid eye movement, muscle twitch and active respiration/heat beat, indicating high activity in the forebrain. In the meantime, associative memory cells for specific events, which have been frequently thought in daytime, are activated. Associative memory cells that are intensively activated in daytime lead to the coactivity-dependent positive cycle of their recruitment and upregulation. So, these events are playbacks. As the reverse relationship between the upregulation of associative memory cells and the requirement of consciousness, associative memory cells with large population and upregulated function due to repeated learning and thinking can be activated under incomplete consciousness condition, such that playback events are incompletely identical to realistic ones ^7^. As the playbacks can be recalled and stated, associative thinking and logical reasoning (the integration of endogenous signals) based on primary and secondary associative memory cells can be fulfilled under incomplete consciousness ^8^. This viewpoint is granted by an observation that temporal sequences of place cell activities in a novel spatial experience are detected during the resting or sleeping period preceding the experience. This preplay occurs in the disjunction to sequences of replay in a familiar experience. These results suggest that internal neuronal dynamics during resting or sleep organize cellular assemblies into temporal sequences that contribute to encode a relevant novel experience in the future ^210^.

Furthermore, images, odors, tastes and events are presented by word-based language in associative thinking and logical reasoning. In initial learning, the sensations, perceptions and events are associated to their correspondent word descriptions, such that associative memory cells for encoding these processes and word descriptions have been recruited. Once these processes are recalled in the sequential playbacks, their word descriptions in these associative memory cells are initiated to substitute the complicated images and events, which is the requirement of speeding up memory retrieval and cognition. The substitution of words to images and events is realized based on the recruitment of more associative memory cells and their upregulations in coactivity-dependent positive cycle manner by repeated practices. However, if words and these processes are associated improperly, the corrections of these associations are difficult because of the presences of these recruited synapse innervations, associative memory cells and their circuits ^7^.

## Associative memory cells in pathology

The integrative storage and the reciprocal retrieval of the associated signals are critical for the bidirectional alertness and prediction in the life. Based on primary and secondary associative memory cells as well as their multi-grade integrations ^7^, one signal will induce the recall of its associated signals, or the other way around, as well as the signals learned from one modality are recalled through the conversion into another modality. Individuals are able to fulfill logical reasoning and associative thinking as well as to predict future events in forward and backward manners. Furthermore, associative memory cells in each of the coactivated brain regions encode the associated innate signal and newly learnt signal, as well as each of the associated signals is stored in multiple brain areas, which largely reduces the chance of memory loss ^8, 11^. The storage of multiple signals in an associative memory cell strengthens the efficiency of memory retrieval ^9^. The storage of multiple signals in a cortical area and the recall of one signal triggered by multiple signals will enable these individuals to strengthen their abilities in memory retrieval and well-organized cognitions. In these regards, the deficit of associative memory cells in their morphology, functions and local environment declines memory retrievals and cognitions as well, which are usually associated with neurological diseases and psychiatric disorders.

It is widely accepted that the normal consciousness and well attention are important for memory formation ^33, 211, 212^, which can be explained by associative memory cells and their features ^7^. With the arousal system to maintain wakefulness and the activation of recruitment-ready neurons by the topic cues in attention call, their activation and activity make them to be able to encode the associated signals. These recruited associative memory cells under the wakefulness condition will grant individuals to identify themselves and environmental objects, which constitute consciousness. On the other hand, the consciousness based on wakefulness and memory supports the activation and activity of associative memory cells to execute activity-dependent positive cycle in their refinement and recruitment, so that more associative memory cells are recruited and impressive memory is formed in the mind. Therefore, the deficit of associative memory cells will make consciousness to be obscure.

Psychological disorders, such as anxiety, depression and even schizophrenia, are accompanied by unusual memory ^143, 213^. For instance, fear memory induced by acute stress is often associated with anxiety ^202, 214^. Stimulations of engram cells through an optogenetic approach in the hippocampus activate fear memory recall and anxiety ^22^. Memory regarding the outcomes of chronic mild stresses are associated with depression-like behaviors ^215, 216^. On the other hand, the activation of positive memory traces by optogenetic methods in the amygdala suppresses depression-like behaviors ^139^. These data indicate that the formation of associative memory cells induced by different patterns of abnormal stimulations can lead to psychological disorders, i.e., acute severe stresses recruit associative memory cells relevant to fear memory and anxiety, and chronic mild stresses recruit associative memory cells related to negative memory and depression ^214, 215^.

The proper coactivation of active neurons makes them to be recruited as associative memory cells ^7, 11^, and the activity-dependent upregulation of associative memory cells facilitates the integrative storage of associated signals ^7, 14, 17, 161, 162^. These processes constitute the coactivity-dependent positive cycle in the recruitment and refinement of associative memory cells, such that more associative memory cells will be recruited. However, the further upregulation of associative memory cells, such as the dysfunction of GABAergic neurons in schizophrenia and epilepsy ^217, 218^, allows associative memory cells to be overly and widely activated. The overly upregulation of associative memory cells in sensory cortices will lead to hallucination. The overly upregulation of associative memory cells in cognition- and emotion-related brain areas leads to illusion ^7^.

The efficiency of learning and memory decays in age-relevant manner ^219, 220^. There is a bell-shaped pattern in the efficiency of associative learning and memory ^11^. In terms of cell mechanisms, synaptic potentiation matures during postnatal development ^180^, and neuronal excitability in cortical neurons is upregulated until a plateau level at postnatal weeks 3–4 ^155^, which matches dynamical changes in associative memory well ^11^. Neural plasticity and associative memory cell recruitment in postnatal development constitute the coactivity-dependent positive cycle in the recruitment and refinement of associative memory cells, such that more associative memory cells are recruited to increase the efficiency of learning and memory ^161, 162^. In older mammalians, the accumulations of insoluble β-amyloid and phosphorylated tau-proteins in the brain influence axon prolongations and synapse formations ^9, 73^ to suppress the recruitment and upregulation of the associative memory cells, to silence active associative memory cells and/or to deteriorate those recruited associative memory cells for the memory deficit ^7, 8, 221^. On the other hand, the activity of associative memory cells can strengthen the coactivity-dependent positive cycle in the recruitment and refinement of associative memory cells, which prevents the conversion of soluble β-amyloid into its insoluble form and promotes the clearance of β-amyloids by associative memory astrocytes ^7, 11^. A current report supports this point in that light and sound stimulations coordinately reduce the accumulation of β-amyloid ^222^.

In age-related neurodegeneration, such as Alzheimer’s disease, insoluble β-amyloid may be accumulated differently in various brain areas. For instance, the optogenetic activation of engram cells, which have a lack of increased synaptic strength and dendritic spines under protein synthesis inhibition-induced amnesia, leads to memory retrievals ^140^. The optogenetic activation of hippocampal engram cells leads to memory retrievals in mice, though they show the amnesia under the condition of using natural recall cues in the transgenic mouse model of early Alzheimer’s disease ^137^. In addition to the indication about the wide distribution of memory traces for signal storage and retrieval, these results suggest that areas involved in natural memory retrieval are dominantly impaired by the deposition of β-amyloid, rather than memory trace cells, as well as that areas responsible for memory retrieval are not specific for a given memory. In this regard, synapse connections from associative memory cells to memory-output cells should be strengthened in the early stage of Alzheimer’s disease ^7, 14, 31^.

In terms of memory maintenance versus extinction, the recruitment and refinement of associative memory cells are not significantly declined, but the activity of memory-output neurons in the motor cortex is lowered ^14, 31^. The sustained presence of associative memory cells as well as the recruitment of more associative memory cells in repeated brain activities confer memorized signals to be retrieved in lifespan, in which the information can be retrieved as long as their innervations onto memory-output neurons successfully drive the latter to be functionally active. It is noteworthy that memory retrievals show different patterns in spontaneous, cue-induced and realistic object-triggered manner with the ages. For instance, spontaneous retrievals often occur in child stage or brain excitation, the cue-induced retrievals usually occur in young and adult, the real object-induced retrievals occur in senior individuals. In addition, when the brain is highly excited in many areas, such as euphoria perception, extreme fear and strong stimulations, more associative memory cells are recruited through their mutual innervations, so that impressive memory and spontaneous recalls to these experiences are generated in lifespan ^14, 31^. It is difficult to remove the newly formed synapse innervation and the recruited associative memory cells to relieve fear memory and addiction. Alternative ways are the avoidance of fear stimuli and the induction of happiness in order to rebalance these two states and weaken fear memory, since the lack of uses in neural circuits related to fear memory, especially from associative memory cells to memory-output neurons, may drive them to be functional silence. In the brains of individuals with history of substance abuse or addiction, primary and secondary associative memory cells relevant to these events are recruited in large amount and in extensive areas under euphoria condition, leading to potential relapses in their lifetime ^8^. Strategies to weaken the addiction in these individuals include the avoidance of the environment cues associated with substance abuse to reduce the output of the relevant associative memory cells, as well as the establishment of alternative happiness to recruit associative memory cells that innervate memory-output cells through competition with the innervations from addiction memory cells, so that the rebalance of these two states strengthens memory-output pathway for happiness ^7^.

## Data availability

No data is associated with this article.
