# Supplementary material for: Searching basic units in memory traces: associative memory cells
Source: F1000Res. 2019 Apr 12;8:457. [Version 1] doi: 10.12688/f1000research.18771.1 (PMC6509747; doi:10.12688/f1000research.18771.1)
Supplement: Peer review history

# Peer review: Reviewer response for version 1

**DOI:** 10.5256/f1000research.20564.r47195

**Published:** 2019-05-08

**Authors:** Hongxin Dong

**Affiliations:** 1 Department of Psychiatry and Behavioral Sciences, Feinberg School of Medicine, Northwestern University , Chicago, IL, USA

In the review, Dr. Wang has significantly expanded discussion on associative memory cells in the brain from the molecular and cellular levels to functional outcomes. The proposed model for learning and memory was supported by work from the author’s own laboratory, as well as from accumulated literature. This is a timely update that will help reveal novel mechanisms in learning and memory formation and loss, which may help to advance understanding of memory deficits in neurodegenerative disorders, such as Alzheimer’s disease.

 In addition to general comments above, the following some suggestions that the author may consider for the future studies.

1. The author has simply discussed the possible relationship between associative memory cells and affective disorders such as anxiety and major depression. It sounds interesting but lacks experimental support. I would suggest the author to give more literature view or conduct some proof-and-concept experiments on this aspect the future.
2. Although the author intends to establish that basic units of memory traces, or engrams, are associative memory cells named by author’s laboratory. It would be more supportive if the author makes the systemic and comprehensive comparisons between cell assemblies in memory traces and associative memory cells. It seems to me that associative memory cells encode the storage of multiple signals, which has not been emphasized by the studies of engrams. As the author states that almost all of learning activities are associative learning, the units to memorize particular associative signals are called as associative memory cells is straight forward. Has the author thought to make a mutual agreement in that associative memory cells and engrams are presenting the same cell ensemble in relevance to memory formation?
3. The theory of associative memory cells may apply in psychology, although numerous terms and theoretical models about learning and memory have been suggested in this field. Hopefully the author and the team would provide more evidence and information to support this theory.  

I have read this submission. I believe that I have an appropriate level of expertise to confirm that it is of an acceptable scientific standard.

# Peer review: Reviewer response for version 1

**DOI:** 10.5256/f1000research.20564.r47193

**Published:** 2019-04-23

**Authors:** Jian-Guo Chen

**Affiliations:** 1 Laboratory of Neuropsychiatric Diseases, Institute of Brain Research, Collaborative Innovation Center for Brain Science, Huazhong University of Science and Technology (HUST), Wuhan, China; 2 Department of Pharmacology, Tongji Medical College, Huazhong University of Science and Technology (HUST), Wuhan, China

This paper by Wang has systemically reviewed cellular units of engrams, presumably associative memory cells. In addition to the history and research approach about engrams, author presents major features of associative memory cells at primary and secondary levels, as well as their impacts in psychology and pathology. The concepts about associative memory cells and their circuits are renewing our understanding to various patterns of learning and memory. This is one of the best reviews in the topic of memory traces, which I would like to approve its publication in F1000Research without delay. I have a few suggestions for the author to move forward in future study:

1. Three diagrams assume neural circuits for associative memory cells to execute memory formation and retrieval, which are mainly based on the studies by author’s group in sensory modalities including somatosensory and olfactory cortices, as well as their downstream regions, the prefrontal cortex and hippocampus. In order to have these diagrams to be generalized truth, the author should also conduct his studies in other areas, such as the visual cortex and auditory cortex.
2. Memories to visual images are mostly complicated in the integration of spatial and temporal information, in comparison with other modalities. Although the author has assumed potential processes for the integration and memory of visual images, I feel that the hypothesized diagram is not perfect and remains to be experimentally tested.
3. The author should design experiments to examine how associative memory cells become dysfunctional under pathological conditions in relevance to memory deficits or amnesia, and are influenced by abnormal internal environments, such as Alzheimer’s disease and Parkinson’s disease.

I have read this submission. I believe that I have an appropriate level of expertise to confirm that it is of an acceptable scientific standard.

# Peer review: Reviewer response for version 1

**DOI:** 10.5256/f1000research.20564.r47192

**Published:** 2019-04-18

**Authors:** Ru-Rong Ji

**Affiliations:** 1 Department of Anesthesiology, Duke University Medical Center, Durham, NC, USA

This is a timely and well written review on associative memory from an expert. The author has been working on this topic for many years, and this extensive review is a very nice summary of the field. All three figures are well presented. I also have a few comments:

1. The manuscript will be greatly improved by including a table or a chart to show historic perspective (e.g., milestones) of the concept development by different groups.  
2. It is interesting to discuss specific miRNAs involved in this process. It will help the readers if the author can include an illustration or a figure to show how specific miRNAs regulate associative memory via specific molecular targets. Overall, discussion on molecular mechanisms of associative memory could be strengthened.
3. People often say “no pain, no gain” ^1^. Is this related to associative memory?   
4. The author discussed some pathological conditions. After anesthesia, there is cognitive decline, such as delirium, especially postoperative delirium in elderly. Is this a related pathological condition?    

I have read this submission. I believe that I have an appropriate level of expertise to confirm that it is of an acceptable scientific standard, however I have significant reservations, as outlined above.

## References

[1] JiRRKohnoTMooreKAWoolfCJ: Central sensitization and LTP: do pain and memory share similar mechanisms?. *Trends Neurosci*.2003;26(12) : 10.1016/j.tins.2003.09.017 696-705 10.1016/j.tins.2003.09.017 14624855
